# Microfluidic Integrated Organic Electrochemical Transistor with a Nanoporous Membrane for Amyloid-β Detection

**DOI:** 10.1021/acsnano.0c09893

**Published:** 2021-03-30

**Authors:** Anil Koklu, Shofarul Wustoni, Valentina-Elena Musteata, David Ohayon, Maximilian Moser, Iain McCulloch, Suzana P. Nunes, Sahika Inal

**Affiliations:** † Biological and Environmental Science and Engineering (BESE), Organic Bioelectronics Laboratory, King Abdullah University of Science and Technology (KAUST), Thuwal 23955-6900, Saudi Arabia; ‡ Advanced Membranes and Porous Materials Center, KAUST, BESE, Thuwal 23955-6900, Saudi Arabia; § Department of Chemistry, University of Oxford, Oxford OX1 3TA, United Kingdom; ⊥ Physical Science and Engineering Division, KAUST Solar Center (KSC), KAUST, Thuwal 23955-6900, Saudi Arabia

**Keywords:** amyloid-β, organic electrochemical transistor, microfluidics, isoporous membrane, biosensors

## Abstract

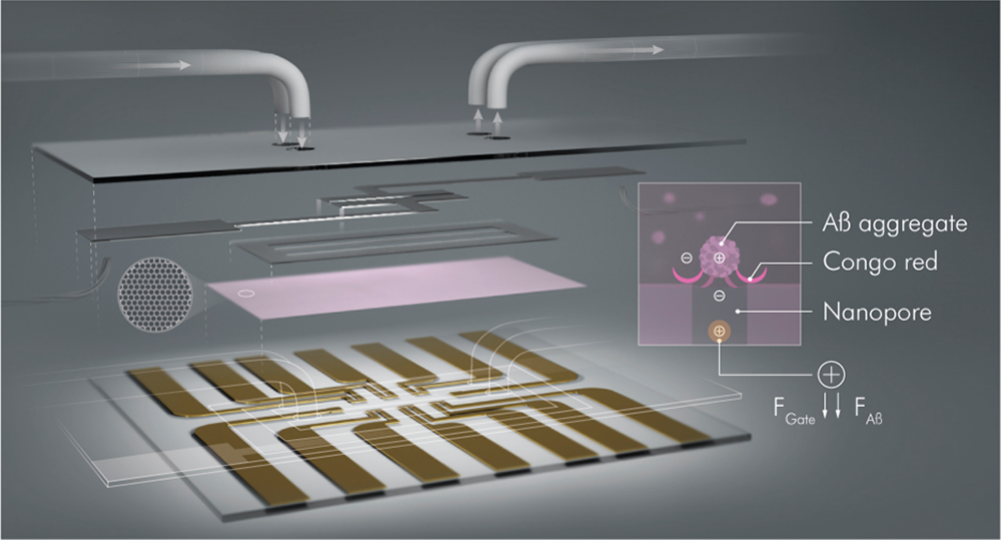

Alzheimer’s disease (AD) is a neurodegenerative disorder associated with a severe loss in thinking, learning, and memory functions of the brain. To date, no specific treatment has been proven to cure AD, with the early diagnosis being vital for mitigating symptoms. A common pathological change found in AD-affected brains is the accumulation of a protein named amyloid-β (Aβ) into plaques. In this work, we developed a micron-scale organic electrochemical transistor (OECT) integrated with a microfluidic platform for the label-free detection of Aβ aggregates in human serum. The OECT channel–electrolyte interface was covered with a nanoporous membrane functionalized with Congo red (CR) molecules showing a strong affinity for Aβ aggregates. Each aggregate binding to the CR-membrane modulated the vertical ion flow toward the channel, changing the transistor characteristics. Thus, the device performance was not limited by the solution ionic strength nor did it rely on Faradaic reactions or conformational changes of bioreceptors. The high transconductance of the OECT, the precise porosity of the membrane, and the compactness endowed by the microfluidic enabled the Aβ aggregate detection over eight orders of magnitude wide concentration range (femtomolar–nanomolar) in 1 μL of human serum samples. We expanded the operation modes of our transistors using different channel materials and found that the accumulation-mode OECTs displayed the lowest power consumption and highest sensitivities. Ultimately, these robust, low-power, sensitive, and miniaturized microfluidic sensors helped to develop point-of-care tools for the early diagnosis of AD.

Dementia is one of the most severe neurodegenerative disorders. The leading cause of dementia in adults is Alzheimer’s disease (AD). AD accounts for more than 80% of all dementia cases globally and costs $290 billion for health care alone, and the number of people forecasted to be affected by this condition by 2050 is 115 million.^1^ Some drugs decelerate AD progression and provide symptomatic relief, but they are usually applied too late to be effective due to the difficulty in diagnosing AD in the early stages. Early diagnosis is expected to minimize the risk of suffering from AD by one-third.^2^ AD diagnosis in clinical setting mainly relies on cognitive tests, sometimes combined with methods that screen for neurotoxic AD-related biomarkers in the brain. Although there is a debate on the identification of AD-related biomarkers (such as amyloid-β (Aβ), tau, phosphorylated tau, neurofilament light chain, vinisin-like protein, neuron enolase, and glial activation),^3^ most studies suggest that the aggregation of toxic senile Aβ peptides into large plaques/precursor proteins is the major pathological hallmark of AD.^4^ The hypothesis suggests that Aβ aggregation starts with the formation of Aβ oligomers, transforming into (proto)fibrils and ultimately leading to plaques.^5^ These aggregates, typically with a diameter of 15–400 nm, deposited in the brain are thought to hinder the communication between neurons, causing their death and blocking key processes that underlie memory and learning.^6^

Neuroimaging techniques are the most popular to monitor Aβ aggregates in the brain.^7−10^ However, these techniques require sophisticated instrumentation in a clinical setting, tedious preparation steps, expensive labels, and long operation times. Optical probes also possess some other limitations, such as a lack of specificity and difficulty detecting concentrations below a few hundred nanomolar, while picomolar range detection is essential for early diagnosis.^4^ AD hallmarks can also be screened *in vitro* using patients’ bodily fluids such as cerebrospinal fluid (CSF) and blood. While CSF sampling *via* lumbar puncture is invasive, a blood-based analysis may facilitate the minimally invasive assessment of AD patients. Specifically, Aβ is found in very small quantities in patients’ blood (25–85 pg mL^–1^), and this may indicate that Aβ is above a certain concentration in CSF and sheds into the vasculature.^11^ Techniques like capillary electrophoresis–mass spectrometry,^12^ calorimetry,^13^ enzyme-linked immunosorbent assay (ELISA),^14^ surface plasmon resonance,^15^ electrochemiluminescence,^16^ and quartz crystal microbalance^17^ have been used for Aβ detection in serum. Yet, most of these methods are time-consuming, not portable, expensive, and labor-intensive. Simple, rapid, and affordable tools for detecting a potential AD biomarker in patient serum can be vital in primary care settings for disease diagnosis, especially for patients in low-income countries where access to hospitals with expensive instrumentation and screening assays is limited.

Electrical methods offer an alternative for Aβ detection in plasma.^4,18^ Most of the early electrochemical sensors for Aβ recorded oxidation signals from protein’s electroactive residues but suffered from a low signal quality, especially when the nonspecific adsorption of serum proteins occurred on electrode surfaces.^19,20^ Recent studies integrated the electronic transducers with specific recognition units such as anti-Aβ antibodies or neuronal receptors, which bind to Aβ.^19−22^ For example, Zhou et al. developed an antibody–aptamer sandwich assay with an Aβ detection limit of 100 pM.^23^ To reach such sensitivity, the assay involved a carboxyl graphene electrode functionalized with an Aβ-antibody and gold nanoparticles comprising an aptamer-redox molecular bioconjugate. Kim et al. used an anti-Aβ antibody immobilized interdigitated electrode.^24^ The platform measured Aβ monomer concentrations as low as 0.1 pg/mL in clinical samples but had to first process serum to dissociate the aggregates. Hideshima et al. integrated Congo red (CR) molecules, which have a strong affinity for Aβ aggregates, on the gate electrode of a field-effect transistor with a detection limit of 100 pM.^22^ We have recently demonstrated the potential of organic electrochemical transistors (OECTs) for Aβ aggregate detection.^25^ The OECT channel was made of a conducting polymer, poly(3,4-ethylenedioxythiophene) doped with polystyrenesulfonate (PEDOT:PSS), separated from the gate electrode, and the electrolyte with an isoporous membrane functionalized with CR. As CR units captured Aβ aggregates, the OECT characteristics changed. This detection scheme has a particular advantage compared to other electronic sensors: the performance is not limited by the solution ionic strength nor does it rely on Faradaic reactions or conformational changes of charged receptors. However, a multi-scale engineering approach is required to fully exploit these features and go beyond the proof-of-concept demonstration toward real-world testing conditions.

In this work, we devised an OECT platform comprising a nanoporous membrane and integrated with a microfluidic channel (**μf-OECT**) for Aβ aggregate detection in human serum (Figure 1). By advancing the OECT technology on several levels, we maximized device performance while overcoming several obstacles. First, PEDOT:PSS is an inherently doped polymer, meaning that the OECTs operate in depletion mode. The high OFF currents and gate voltages applied to keep the device in its off-state increase power consumption, a disadvantage for integration, and pose a risk to material stability for long-term use. Here, we used three types of (semi)conducting polymers in the channel, allowing us to compare the sensor performance of OECTs operating in two distinct modes, i.e., accumulation and depletion modes. We show that the accumulation-mode OECTs reach a higher sensitivity and range of detection than the depletion-mode devices while exhibiting lower power consumption. Second, a large electrolyte reservoir necessitates a large sample volume. A large volume is not practical for human samples and leads to long incubation times of the sensing surface with the sample to compensate for the long diffusion times of the analyte (*t*_*D*_ = *L*^2^/ *D*, where *L* is the reservoir height (analyte solution) and *D* is the diffusion coefficient of analyte). By combining the OECT with the microfluidic system, we precisely processed minute amounts of fluids. Requiring only 1 μL of human serum for incubation and the same amount of electrolyte for operation, microfluidics integration reduced the sample incubation time to 1 h and the sample/electrolyte volume from 100 to 1 μL. The use of microfluidics also lowered the limit of detection from nanomolar to femtomolar and extended the detection range (2.21 fM–221 nM), while enabling standardization. Moreover, microfluidics allowed us to simultaneously obtain control measurements by compartmentalizing sensor components and sample solution, increasing the accuracy of recordings. Lastly, since we physically separate the detection layer and the electronics, the electronic components no longer need to be chemically modified to anchor biorecognition units, making the device design less stringent and more stable. This simple detection strategy obviates the use of reference electrodes or electroactive labels that many electronic immunosensors rely on. Thus, our device design aids in developing low-cost, high throughput, portable, and stable electronic tools for the diagnosis of biomarkers of diseases such as AD.

**Figure 1:**
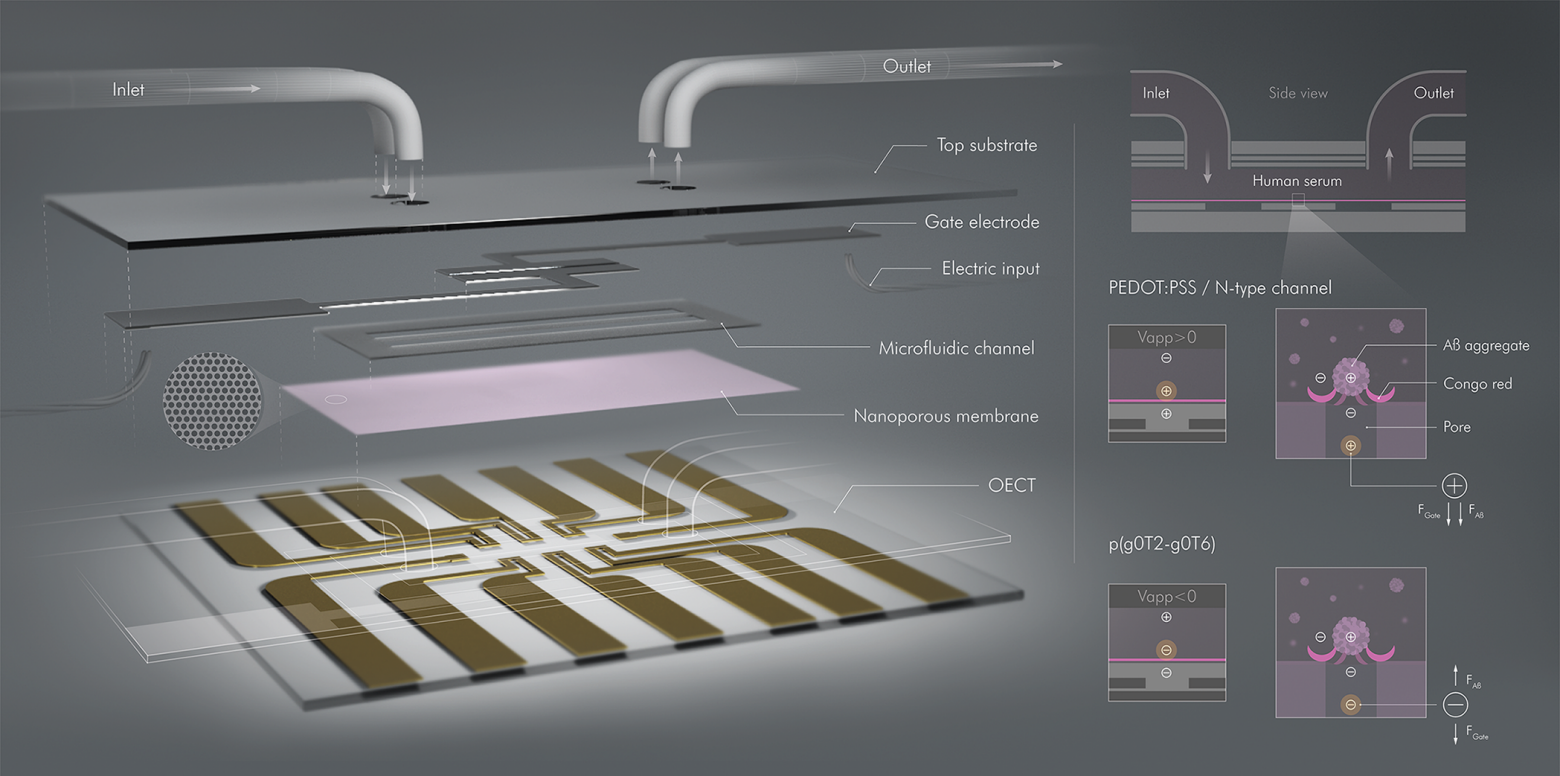
Schematic of the μf-OECT for Aβ detection (left panel) and the sensor operation principle (right panel). The components of the device are assembled into the final form, as illustrated in its cross-sectional view (top right). In between the OECT channels at sensor base and the gate electrodes on the top substrate, we placed the nanoporous membrane functionalized with CR. The microfluidic channel retains 1 μL of sample solution over six identical OECT channels operated by two top gate electrodes. Bottom right images zoom in on one membrane pore/channel interface and illustrate the ion flux from the gate electrode toward the channel (made of PEDOT:PSS, an *n*-type semiconductor (p(C_6_NDI-T)), or the *p*-type semiconductor, p(g0T2-g6T2)). The positively charged Aβ aggregates captured by the CR of the membrane modulate the gate voltage imposed on the channel.

## Results/Discussion

### Characterization of the Isoporous Membrane

**Figure 2:**
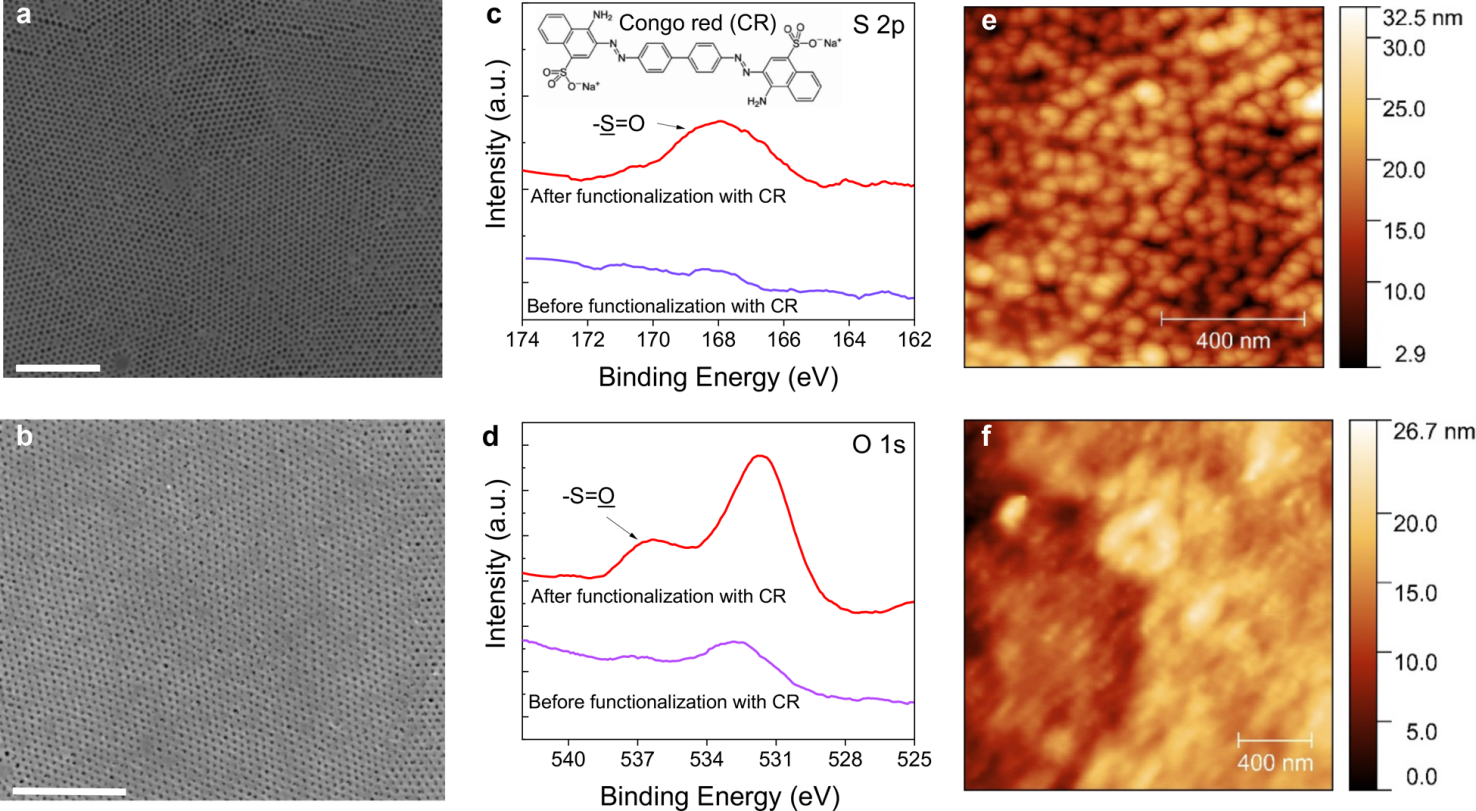
Characterization of the functionalized isoporous PS-*b*-P4VP membrane. FESEM images of the (a) pristine membrane and (b) CR-functionalized membrane. The scale bars are 1 μm. XPS (c) S 2p and (d) O 1s analyses of the membrane before and after CR functionalization. In c, the membrane was monitored after APTES treatment. AFM images of the CR functionalized membrane (e) before and (f) after Aβ aggregate binding.

The CR-functionalized isoporous nanostructured membrane is the essential component of the μf-OECT sensor, which we characterized using field emission scanning electron microscopy (FESEM), X-ray photoelectron spectroscopy (XPS), and atomic force microscopy (AFM). We prepared the PS-*b*-P4VP membrane using self-assembly copolymerization and nonsolvent induced phase separation (see the Methods/Experimental section and the Supporting Information).^26,27^ A comprehensive investigation of a similar membrane morphology has previously been reported.^28^ To quantify the porosity at different layers of depth, the authors performed a segmentation and applied an algorithm based on the 3D characterization of a focused ion beam and serial block face SEM images. The membrane showed an asymmetric profile with a gradient of porosity increasing from the top to the bottom. Our membrane, prepared using the same protocol, contains uniform nanopores on the surface with an average diameter of 41 ± 2 nm and a pore density of 188 pores per square micrometer, as evaluated from the SEM images of the surface (Figure 2a). We treated the membrane with the CR ligand binding specifically to Aβ aggregates but not to the Aβ peptide.^29^ We chose CR as the recognition unit as it selectively binds to the β-pleated sheet structure of amyloid fibrils^30^ and is durable, easy to conjugate, and less expensive compared to protein-based binding domains. The FESEM image of the membrane after CR functionalization is shown in Figure 2b, revealing a reduction in the average pore diameter (down to 23 ± 6 nm).

The high-resolution S 2p and O 1s XPS spectra of the membrane surface showed significant changes in the chemical composition as a result of CR immobilization. First, in the S 2p spectrum of the CR-functionalized membrane, we captured a peak characteristic to a sulfur atom (Figure 2c). Second, the O 1s XPS spectrum of CR-functionalized membrane displayed additional peaks at 535–537 eV, which were not observed in the APTES-modified surface (Figure 2d). These sulfur and oxygen peaks are a fingerprint of the -SO_3_ functional group of the CR molecules. Hence, XPS characterization proves that the membrane surface has been successfully modified with CR molecules. We next took AFM images of the CR-functionalized membrane after incubating it with a solution of Aβ aggregates (2.21 nM in PBS). The AFM image in Figure 2f reveals that Aβ aggregates clogged the open pores of the membrane (Figure 2e). The morphology of these aggregates was further verified with FESEM imaging, suggesting a globular structure with a diameter of ranging from ca. 40 nm to few hundreds of nanometers (Figure S1), consistent with reported values in the literature.^6^ We also estimated the hydrodynamic radius of the Aβ aggregates in the solution using a particle analyzer and found it to be around 198 nm (in 2.21 nM) and 171 nm (in 2.21 pM and 2.21 fM) (Figure S2).

### Performance of the μf-OECT for Detecting Aβ Aggregates

**Figure 3:**
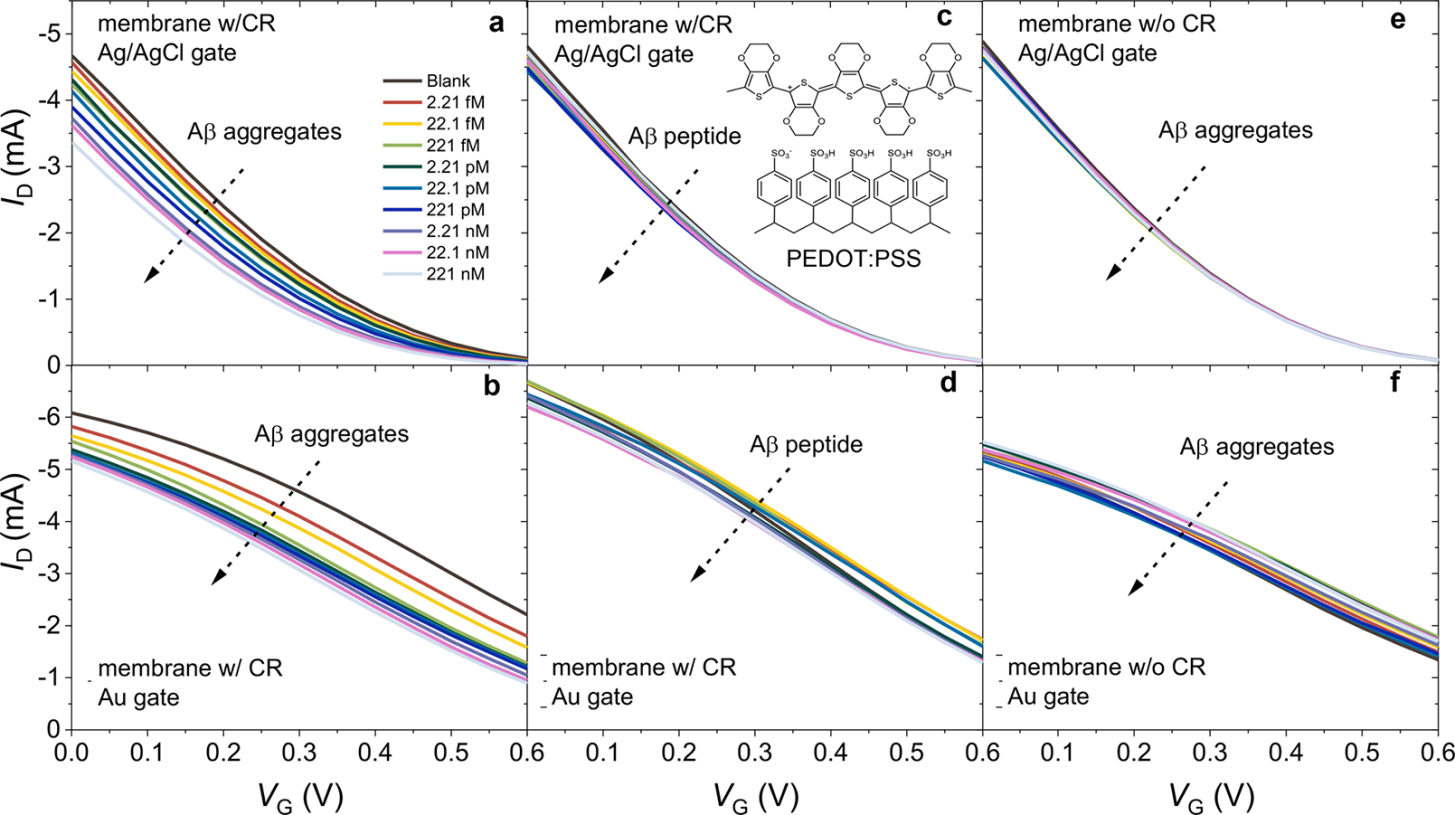
Transfer characteristics of (a, c, and e) Ag/AgCl and (b, d, and f) Au electrode gated μf-OECTs comprising PEDOT:PSS in the channel. The arrows represent the increase in Aβ aggregate (a and b) and Aβ peptide (c and d) concentrations in PBS from 2.21 fM to 221 nM. The inset of c depicts PEDOT:PSS’ chemical structure. The transfer characteristics of the pristine membrane (no CR functionalization) integrated devices are shown in e and f. The *V*_D_ was −0.6 V for all devices. The sample solution in the fluidic channel was 1 μL.

The OECT is an electrolyte gated transistor that contains a (semi)conducting polymer film in the channel. As a gate voltage is applied, electrolyte ions penetrate the polymer bulk and change its doping state, hence the channel conductivity. The volumetric ionic-electronic charge coupling endows OECTs with high transconductance; thus, while converting ionic fluxes in the electrolyte into an electrical output, the device amplifies these signals on the site.^31^ When functionalized with (bio)recognition units, these high-gain devices can thus sense various species such as metal cations,^32^ metabolites,^33^ pathogens,^34^ and proteins.^35,36^Figure S3 shows the current–voltage characteristics of a typical PEDOT:PSS-based μf-OECT developed in this work. A positive voltage at the gate electrode (*V*_G_) drifts electrolyte cations toward the PEDOT:PSS channel to compensate for the extracted holes, while the anions are attracted to the gate electrode. We thus observed a decrease in the channel current, *I*_D_, with an increasing *V*_G_, consistent with the depletion-mode operation. When we integrated the membrane on the channel, the device characteristics did not change, attributed to the permeability of the membrane, allowing for efficient gating of the channel (Figure S3). After we incubated the μf-OECTs with different concentrations of Aβ, we recorded the transfer characteristics using Ag/AgCl or an Au electrode as the gate. Parts a and b of Figure 3 show that, independent of the gate electrode type, *I*_D_ had a continuous decrease with an increase in the Aβ aggregate concentration. Parts c and d of Figure 3 are the transfer characteristics of the neighboring μf-OECTs exposed to Aβ in its peptide form. The negligible change in the current with the peptide confirms the selectivity of the CR toward to the aggregate form of the protein identified with a cross-β structure.^22,37,38^ When the membrane did not contain CR, the device had no sensitivity to Aβ (Figure 3e,f). The current was also not affected as we injected blank PBS solutions multiple times (Figure S4), suggesting high operational stability.

**Figure 4:**
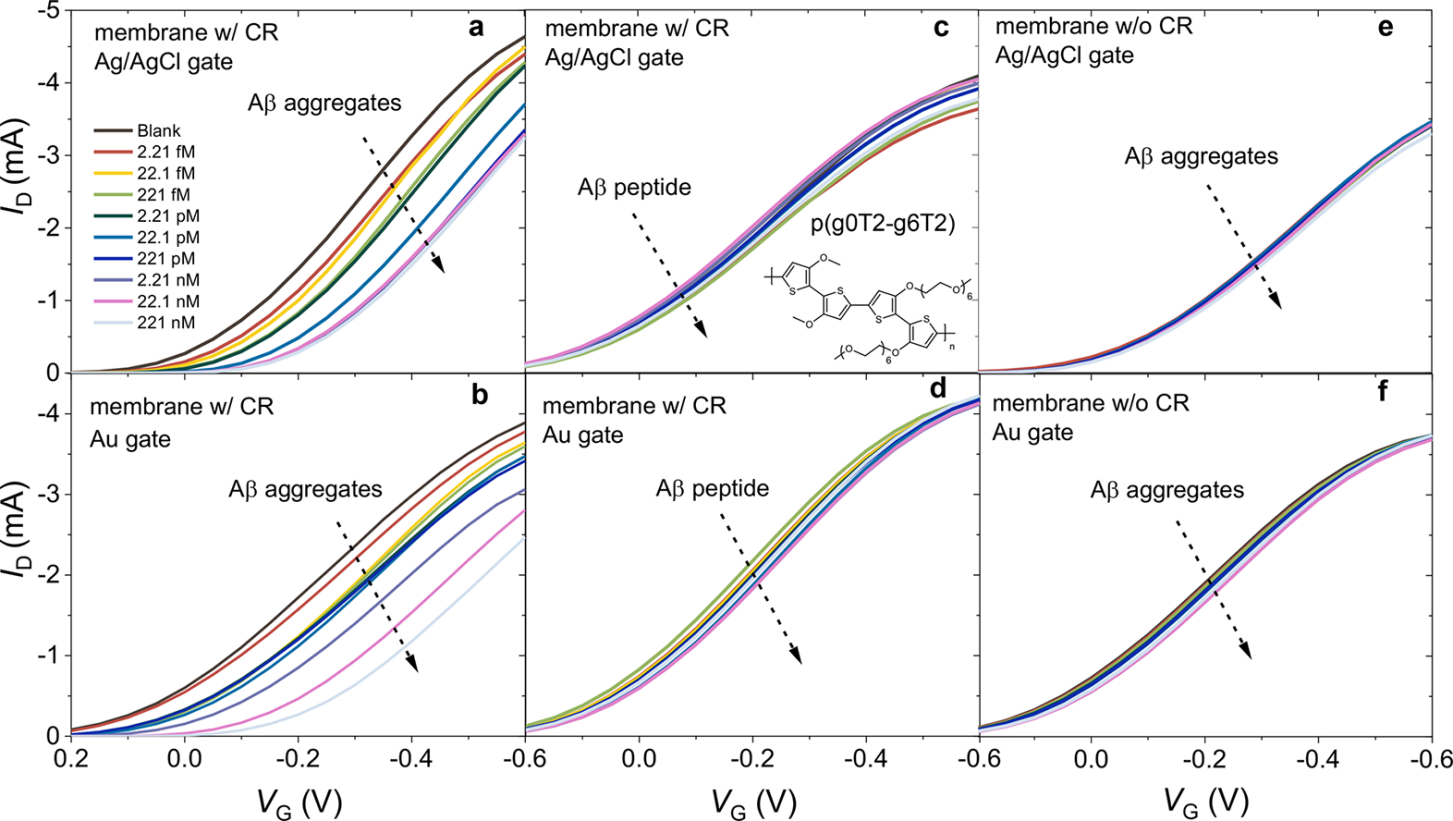
Transfer characteristics of (a, c, and e) Ag/AgCl and (b, d, and f) Au electrode gated μf-OECTs containing p(g0T2-g6T2) in the channel. The arrows represent the increase in Aβ aggregate (a and b) and peptide (c and d) concentrations in the sample solution from 2.21 fM to 221 nM. The inset of c depicts the chemical structure of p(g0T2-g6T2). The transfer characteristics of the pristine membrane (no CR functionalization) integrated devices are shown in e and f. *V*_D_ was −0.6 V for all the devices. The sample solution in the fluidic channel was 1 μL.

Next, we replaced PEDOT:PSS in the channel with the *p*-type semiconducting polymer, p(g0T2-g6T2), and built “accumulation-mode” OECTs. The application of a negative *V*_G_ pushes anions into the p(g0T2-g6T2) film that compensate for the holes injected from the metal contacts, turning the device ON (Figure S5). Such accumulation-mode devices, where the initially OFF channel generates a current upon the application of a gate voltage, are considered more advantageous for biosensing applications due to their low-power consumption at the subthreshold regime, low threshold voltages that avoid parasitic reactions in ambient conditions, and a drastic change in channel current upon the biorecognition event.^36,39−41^ All our devices showed minimal hysteresis with almost identical behavior, as observed from forward and backward voltage scans (Figure S6). Figure S6 shows that the p(g0T2-g6T2) transistors had low OFF currents on the order of 10 μA. While the ON/OFF ratio at gate voltages, which lead to a maximum *g*_m_ in saturation regime, was about 10 for PEDOT:PSS OECTs; this value was 500 for p(g0T2-g6T2) (Figure S6, ON/OFF ratio of p(C_6_NDI-T) OECT is 850). The maximum transconductance (*g*_*m*_ = (∂*I*_D_/∂*V*_G_)) of p(g0T2-g6T2) OECTs was on the order of 10 mS, similar to those of PEDOT:PSS-based ones (Figures S7 and S8). We also investigated the operational stability of our devices by switching them “ON” and “OFF” for 10 s each and recording the *I*_D_ over 180 cycles (Figure S9). PEDOT:PSS retained 84% of its initial current, and accumulation-mode transistors showed a somewhat better stability with an Δ*I*/*I*_0_ of 99%. Parts a and b of Figures 4 show the typical transfer characteristics of these devices when exposed to solutions with various Aβ concentrations and gated with Ag/AgCl and gold electrodes, respectively. As Aβ aggregates bound to the CR functionalized isoporous membrane surface, *I*_D_ decreased for all gate voltages. The current decrease was accompanied by a significant shift in the threshold voltage (*V*_th_) toward more negative values (Figure S10). The devices were stable (Figure S11) and showed excellent selectivity toward Aβ aggregates (Figure 4c,d) endowed by the CR functionalization of the membrane (Figure 4e,f).

**Figure 5:**
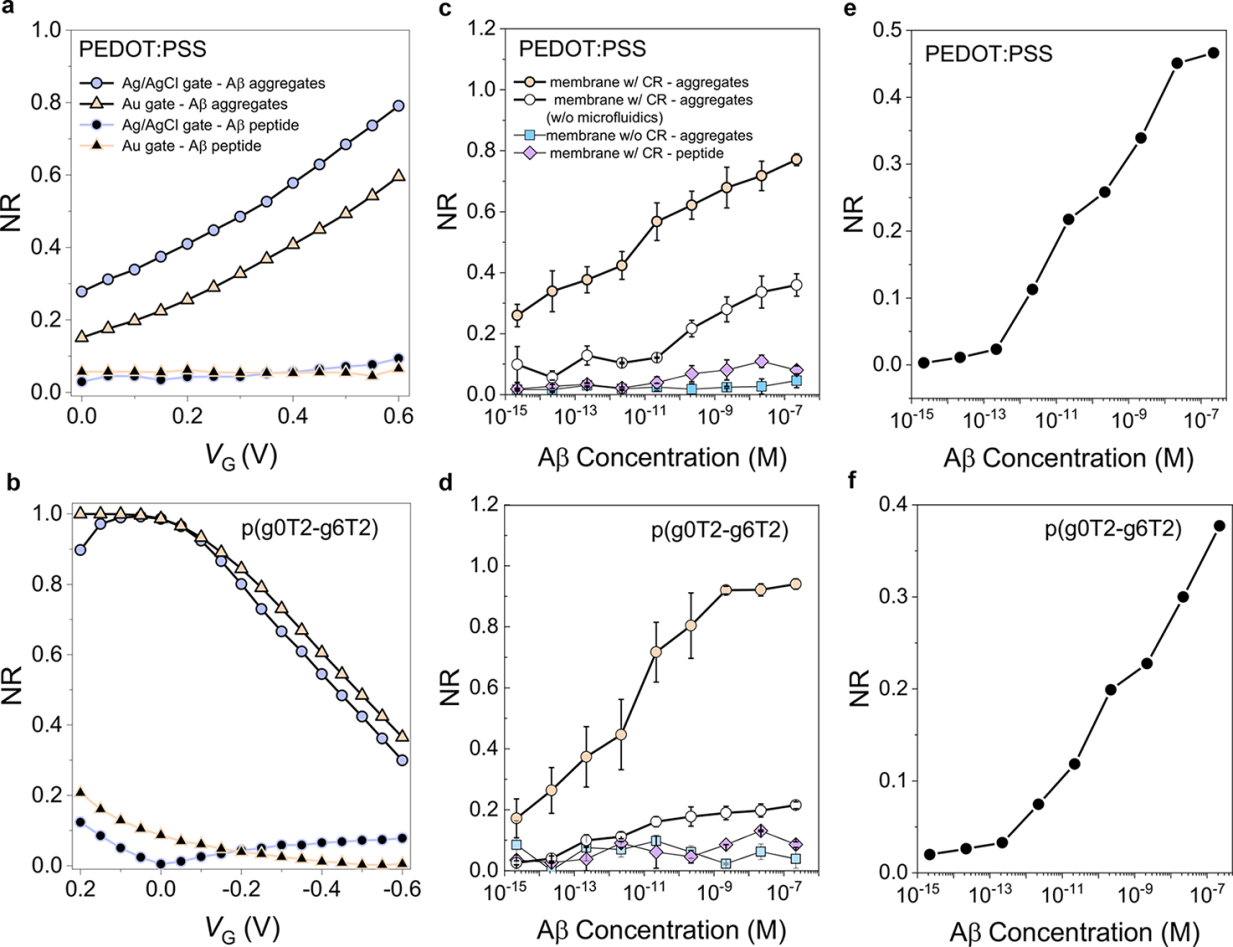
Normalized response (NR) of the sensors with (a and c) PEDOT:PSS and (b and d) p(g0T2-g6T2) channels. In a and b, the recorded response was to a single Aβ aggregate or peptide concentration (221 nM) as a function of *V*_G_ and the devices were gated with Ag/AgCl or Au. In c) and d), the gate electrode was Ag/AgCl. For the microfluidic-free device, a 1 mm thick PDMS well was placed on top of the OECT to confine 100 μL of PBS over an area with a diameter of 1 cm. NR was calculated at *V*_G_ = 0.6 V for PEDOT:PSS and *V*_G_ = −0.1 V for p(g0T2-g6T2) devices. Error bars represent the SD from at least three different channels. *V*_D_ was −0.6 V in all measurements. Panels e and f show the normalized change in impedance magnitude measured at 10 kHz for PEDOT:PSS and p(g0T2-g6T2) films, respectively.

We assessed the sensing performance of our devices by considering the change in *I*_D_ at a given operating condition, normalized by the current measured before the sensing event (i.e., normalized response, NR). Parts a and b of Figure 5 show that, for the binding of 221 nM of Aβ aggregates, the PEDOT:PSS-based devices had the highest NR when operated at *V*_G_ = 0.6 V, while the highest NR was attained at +0.1 V ≤ *V*_G_ ≤ −0.1 V for p(g0T2-g6T2). PEDOT:PSS-based sensors had an NR dependent on the gate electrode type, i.e., Ag/AgCl improved the response compared to Au. On the contrary, the gate electrode played a minor role in determining the NR of p(g0T2-g6T2) sensors. The calibration curves of these sensors are given in Figure 5c,d. Both devices had a broad dynamic range, from 2.21 fM to 221 nM (eight orders of magnitude), adequate for the determination of Aβ in clinical samples.^4^ The p(g0T2-g6T2) devices showed a higher sensitivity compared to PEDOT:PSS, and above 20 fM, the NR was higher. The limit of detection (LOD), calculated according to eq 2, was as low as 100 zM. However, in 1 μL of 2.21 nM Aβ aggregate solution, the number of aggregates was ca. 750 000, which decreased to 1060 in 2.21 pM solution, and one aggregate could be found in a 2.21 fM solution (Figure S7; see the Methods/Experimental section). Accordingly, 1 μL of sample solution in the microfluidic channel may not even contain protein aggregates at a zeptomolar and femtomolar range. We, therefore, evaluated the sensor response to Aβ aggregate concentrations that are lower than 2.21 fM. Our sensor did not show any response to the zeptomolar-to-femtomolar range with the obtained NR values close to those attained for the negative control (Figure S12). We thus conclude that the lowest concentration that can be detected by our OECT sensor is 2.21 fM in PBS.

To evaluate the effect of microfluidics on sensor performance, we recorded these devices’ characteristics, this time using a conventional PDMS well (Figure S13). The microfluidic-free OECTs showed inferior sensing characteristics such as a low NR and sensitivity values compared to those of μf-OECTs (Table 1). Given a specific incubation time, the long diffusion time of Aβ aggregates in this large sample volume inside the PDMS well will limit the interactions between the aggregates and the CR units. The longer diffusion times, in turn, result in lower NR values. On the contrary, the confinement within the μf-OECT improves the sensor response, translated into NR values, which increased at least two times for each protein concentration.

**Table 1:** Characteristics of the OECT Sensors Developed in This Work^a^

			PBS		human serum	
channel	gate electrode	device configuration	c-NR	(sensitivity) (a.u/M^–1^)	c-NR	(sensitivity) (a.u./M^–1^)
PEDOT:PSS	Au	w/microfluidic	22.1 fM	4.57	221 fM	8.39
		w/o microfluidic	22.1 pM	3.17		
	Ag/AgCl	w/microfluidic	2.21 fM	6.60	2.21 pM	14.11
		w/o microfluidic	22.1 pM	6.11		
p(g0T2-g6T2)	Au	w/microfluidic	221 fM	11.34	2.21 pM	11.78
		w/o microfluidic	22.1 pM	5.71		
	Ag/AgCl	w/microfluidic	2.21 fM	12.83	2.21 fM	14.12
		w/o microfluidic	22.1 pM	2.53		

a The channel was made of either PEDOT:PSS or p(g0T2-g6T2), gated with Au or Ag/AgCl electrodes, and operated in after exposure to PBS or human serum. c-NR values were determined as the minimum concentration for which the sensor gives a higher NR than the maximum NR generated by the negative control. Sensitivity was calculated as the slope of the calibration curve (NR vs aggregate concentration).

Electrolyte gated transistors are typically used to transduce and amplify the detection events occurring at a biofunctionalized gate electrode.^42^ Since our sensing mechanism does not rely on potential or Faradaic changes at the gate electrode, we sought to understand the advantage of the OECT configuration over a conventional two-electrode system. Electrochemical impedance spectroscopy (EIS) measurements were performed to screen the effect of Aβ concentrations on the impedance of the microfluidic integrated PEDOT:PSS and p(g0T2-g6T2) channels covered with the functional membrane. We used our in-built Ag/AgCl electrodes as the counter/reference electrode. The impedance spectra were measured, first, in Aβ-free PBS solution and, then, after the membranes were incubated with successive concentrations of Aβ (from 2.21 fM to 221 nM), followed by a rinsing step. As Aβ molecules accumulated on the porous membrane, they hindered ion transport toward the channel, increasing the impedance magnitude particularly at the high-frequency regime (>1 kHz) (Figure S14). The normalized change in impedance magnitude as a function of Aβ concentrations is shown in Figure 5e,f. These values are six orders of magnitude higher than those attained with the OECT configuration. Thanks to the local amplification endowed by the transistor circuitry, the use of transistors rather than a conventional two-electrode configuration results in increased signal-to-noise ratios, translated into elevated sensor sensitivities and improved LOD values.^43^ When we compared the LOD of our μf-OECTs to other prominent Aβ sensors reported, we found it to be lower than those of electrochemical ones^4,44,45^ and similar to those of the optical sensors,^4^ despite the simplicity of our design. Overall, the high transconductance of the OECT, the precise porosity of the membrane, and the compactness endowed by the microfluidic enable the Aβ aggregate detection in 1 μL of human serum samples as low as 3 fM and over a concentration range spanning eight orders of magnitude (femtomolar–nanomolar). The accumulation-mode devices have the added benefit of low operation voltages and device stability.

### Detecting Aβ Aggregates in Human Serum

**Figure 6:**
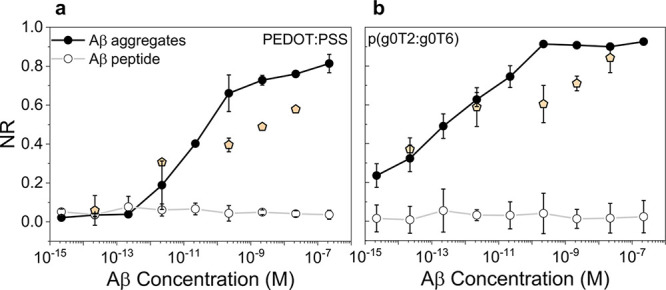
NR of (a) PEDOT:PSS and (b) p(g0T2-g6T2) μf-OECTs to Aβ aggregates or Aβ peptides present at various concentrations in human serum. NR was determined at *V*_G_ = 0.6 V for PEDOT:PSS and *V*_G_ = −0.1 V for p(g0T2-g6T2) μf-OECTs, which were gated with Ag/AgCl. *V*_D_ was −0.6 V for both devices. The orange symbols represent the data obtained upon exposure of the sensors to single Aβ aggregate concentrations in human serum (1 μL). These data were recorded at a fixed gate and drain bias (*V*_G_ = 0.6 V for PEDOT:PSS and *V*_G_ = −0.1 V for p(g0T2-g6T2); *V*_D_ = −0.6 V). Error bars represent the standard deviation (SD) from at least three different channels. See Figure S15 for the results of the same experiments performed with an Au gate electrode.

We next evaluated the sensor performance in the target medium, human serum. Human serum contains various molecules such as glucose, albumin, and cholesterol.^46^ The μf-OECTs detected Aβ aggregates in human serum within a broad range of concentrations (2.21 fM–221 nM) (Table 1). The interference from the serum or Aβ in peptide form was minimal (Figure 6 and Figures S15 and S16). Next, to better emulate real-world screening conditions, we exposed the devices to random Aβ aggregate concentrations. Moreover, instead of sweeping a range of biases, we operated these devices at a fixed gate and drain voltage. The single biasing is convenient to minimize power consumption and measurement times, rendering the device easily adaptable for integration. Both OECTs responded with changes in their output current similar to those obtained during broad-range biasing experiments for concentrations below 0.1 nM (Figure 6). Above 0.1 nM, the NR was scaled with the protein content but the values recorded were lower compared to those in standard calibration curves.

When the PEDOT:PSS-based μf-OECT was exposed to Aβ aggregates, we observed a significant decrease in the channel current. If the membrane pores were blocked by Aβ aggregates captured by CR, we should have, in theory, less cations penetrating the channel, interacting with PSS, and depleting the holes. The impeded ion flow should thus result in an increase in the channel current at all gate voltages rather than a decrease. The *I*_D_ decrease with Aβ capture can be understood by considering the ionic charge that aggregates introduce to the membrane as they accumulate on it. To determine the change in the total capacitance of the system upon protein binding, we analyzed the transfer curves in Figure 3 using the Bernards–Malliaras model (see the Supporting Information).^35,47^Figure S17 shows that the capacitance of the system increases upon binding of the Aβ aggregates, which is possible only if the total charge flowing therein increases. Since Aβ aggregates are positively charged in PBS (pH = 7.4), they repel cations at the pores toward the channel. Hence, the total electrical force acting on the cations during the application of a positive gate bias becomes larger when the aggregates accumulate on top of the pores (Figure 1, right panel). As more proteins are trapped, the reduction in *I*_D_ becomes larger (higher NR). Previous work, which performed EIS measurements to monitor the binding of Aβ aggregates to a receptor immobilized on an electrode, observed a decrease in electrode impedance with an increase in Aβ binding events.^48−50^ Due to their conducting nature (σ = 1.7 S/m),^51−53^ the Aβ binding increased the current density flowing through the electrolyte–electrode interface. In agreement with these studies, we observed a decrease in impedance magnitude recorded at high frequencies with an increase in Aβ concentrations (Figure S14). Contrary to PEDOT:PSS, the aggregate binding does not ease the gating of the p(g0T2-g0T6) channel. In these devices, we applied a negative *V*_G_, which pushes anions into the film. In the presence of the positively charged Aβ-trapped membrane at the channel/electrolyte interface, the number of anions penetrating into the polymer is reduced, decreasing the channel current (Figure 1, right panel).

**Figure 7:**
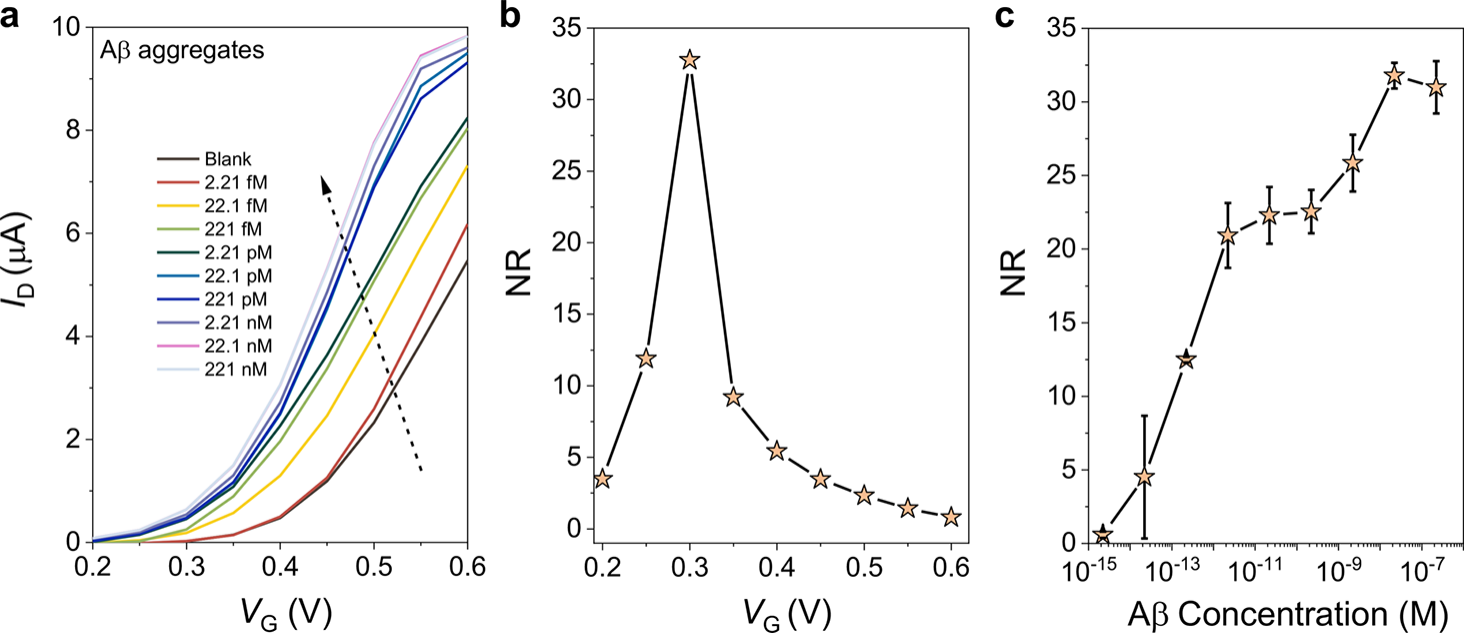
(a) Transfer characteristics of *n*-type p(C_6_NDI-T) μf-OECTs. The arrows represent the increase in Aβ concentration in the sample solution from 2.21 fM to 221 nM. (b) NR of the device to Aβ aggregates (221 nM) calculated at various gate voltages. (c) Calibration curve of *n*-type μf-OECTs for Aβ sensing, reported at a *V*_G_ of 0.3 V. The error bars represent the SD from at least three different channels. All devices were gated with Ag/AgCl, and the *V*_D_ was at 0.2 V.

To validate our hypothesis for the working principle of these sensors, we conducted the same measurements, this time with a μf-OECT comprising an *n*-type semiconducting polymer, namely, p(C_6_NDI-T), in the channel. The *n*-type OECT operates in accumulation mode, i.e., a positive voltage at the gate electrode pushes the cations into the channel, which electrostatically compensate for the electrons injected from the contact. Figure S18 shows the typical output and transconductance characteristics of these devices (see Figurse S6 and S9 for the hysteresis and transient characteristics, respectively). As we incubated the membrane with Aβ aggregates, the *n*-type OECTs showed a significant decrease in the drain voltage where a transition from the linear to saturation regime starts (Figure S18c). Consequently, particularly at the linear regime (*V*_D_ < ca. 0.25 V), the channel current increased for each subsequent injection of solutions with increasing concentrations of Aβ (Figure 7a). The devices showed the maximum response to Aβ when biased at a *V*_G_ of 0.3 V (Figure 7b) and the increase in *I*_D_ scaled with Aβ concentrations (Figure 7c). We note the superior sensitivity of these devices compared to the *p*-type sensors. These results support the working principle of the sensors described above. Aβ binding increases the overall capacitance and amplifies the electrical field imposed on the channel. More cations located in the nanopores are repelled toward the channel, which results in higher *I*_D_ values. Finally, comparing the power consumption of each device type, we found that accumulation-mode devices have a lower power demand when operated at the subthreshold regime, which yields the maximum NR values (Figure S19). The power consumption in PBS is 0.29 mW for PEDOT:PSS (*V*_G_ = 0.6 V, *V*_D_ = −0.6 V), 0.21 mW for p(g0T2-g6T2) (*V*_G_ = −0.1 V, *V*_D_ = −0.6 V), and 0.057 μW for p(C_6_NDI-T) (*V*_G_ = 0.3 V, *V*_D_ = 0.2 V).

To investigate the physical grounds of ion movement on the channel surface, we performed finite element-method-based numerical simulations by solving the Poisson–Nernst–Planck (PNP) equations. A theoretical model describing the physics is included in the Supporting Information. Our experimental findings are consistent with the numerical simulations. The results show that the average electric field intensity always increases in the presence of Aβ aggregates owing to the extra voltage supply. Since the electric field intensity is amplified inside the nanopore, cation transport toward the channel surface becomes more effective, while anions are attracted toward the positively charged Aβ aggregates. We calculated the average concentration of ions on the channel surface for all device configurations 60 s after the application of drain and gate voltages. The average cation concentration increases at the surface of PEDOT:PSS and the *n*-type polymer channel, while the anion concentration decreases at the p(g0T2-g6T2) surface (Figure S20b).

## Conclusions

In this work, we developed microfluidics-integrated microscale OECTs that comprise an isoporous nanostructured membrane. These label-free devices detected Aβ protein aggregates in human serum with a performance exceeding those of several other systems. The membrane was functionalized with CR molecules, which selectively captured the protein aggregates. As the membrane surface was covered with the aggregates, its capacitance changed, which, in turn, modulated the effective gate voltage felt by the transistor channel. The microfluidic channel served as an immunoreaction chamber that led to a significant decrease in analysis time with a minimal sample/reagent utilization compared to microwell technology. We showed the sensor operation principle with three types of channel materials identified by two distinct operation modes, namely, accumulation and depletion. For all devices, the binding event affected the transistor characteristics drastically, i.e., *I*_D_ changed as a function of the captured Aβ aggregates on the membrane. The microfluidic-based OECTs detected the Aβ aggregates in a broad concentration range (from 2.21 fM to 221 nM) in the buffer and human serum samples. The accumulation-mode devices performed superior to the depletion-mode ones due to their low-power demanding nature and higher changes in current output triggered by the binding event. Overall, our simple detection strategy obviates the use of reference electrodes or electroactive labels that many electronic immunosensors rely on and is applicable for a wide range of analytes. The sensor does not involve chemical functionalization of the electronic components, which typically results in their deterioration. Our microfluidic- and nanoporous-membrane-integrated OECT immunosensor enables the real-time, sensitive detection of biomarkers in bodily fluids, and such designs will be useful for the development of portable diagnostic devices.

## Methods/Experimental

### Materials

The block copolymer poly(styrene-*b*-4-vinylpyridine) (P10900-S4VP, 188000-b-64000 g/mol) was purchased from Polymer Source, Inc. (Dorval, Canada). Dimethylformamide, 1,4-dioxane, acetone, 4-chloro-1-butanol, ethanol (EtOH), (3-aminopropyl)triethoxysilane (APTES), glutaraldehyde (GA), Congo red (CR), ethylene glycol (EG), dodecyl benzene sulfonic acid (DBSA), (3-glycidyloxypropyl)trimethoxysilane (GOPS), sodium chloride (NaCl), silver nitrate (AgNO_3_), and phosphate buffered saline (1X PBS, pH 7.4, ionic strength 0.162 M) were acquired from Sigma-Aldrich (Taufkirchen, Germany). The conducting polymer poly(ethylenedioxythiophene):poly(styrenesulfonate) (PEDOT:PSS, PH1000) dispersion was purchased from Heraeus Clevios GmbH (Leverkusen, Germany). The recombinant human Aβ 1–42 peptide (#ab82795) was purchased from Abcam (Cambridge, MA). All aqueous solutions were prepared with ultrapure water (Milli-Q, Millipore). The *p*-type semiconductor, poly(2-(3,3′-bismethoxy)-[2,2′-bithiophen]-5yl)-alt-(2-(3,3′-hexaethyleneglycol monomethyl ether)-[2,2′-bithiophen]-5yl), abbreviated as p(g0T2-g6T2), was synthesized on the basis of the protocols in ref (54). The synthesis of the *n*-type material was based on the protocol we reported in ref (33), and the detailed information is given in the Supporting Information (Figures S21 and S22). Human serum from human male AB plasma was purchased from Sigma-Aldrich (Taufkirchen, Germany).

### Preparation of the Functional Isoporous Membrane

The isoporous membrane was prepared as reported before,^26^ from the block copolymer poly(styrene-*b*-4vinylpyridine), PS-*b*-P4VP, and is detailed in Figure S23. Briefly, the membranes were prepared by block copolymer self-assembly and nonsolvent-induced phase separation. The 4VP block was then quaternized using 4-chlorobutan-1-ol to covalently bind the recognition units on the membrane surface. The membrane was immersed in a 2.5% (v/v) solution of 4-chlorobutan-1-ol in ethanol at room temperature for 24 h. Then, the membrane was pulled out and rinsed with ultrapure DI water. Next, the quaternized membranes were exposed to a 5% (v/v) solution of APTES in water for 30 min, followed by washing with ultrapure water. We then used the bifunctional linker GA, which reacts with the amino groups of the APTES-modified membrane on one end and with CR molecules on the other. For that, the amine-terminated membranes were submerged in 5% (w/v) GA solution for 30 min. Then, the CR was covalently bonded by incubating the membrane in a CR solution (1 μg/mL) for 1 h. Each functionalization was followed by rinsing in PBS and drying by flushing with N_2_ to remove any low molecular weight and unreacted molecules from the surface.

### Preparation of Amyloid-β Monomer and Aggregate Solutions

The Aβ monomers were dissolved in 1X PBS solution containing 0.5% ammonium hydroxide for a final stock solution concentration of 221 nM. This stock solution was diluted using PBS to obtain various Aβ concentrations between 2.21 fM and 221 nM. The Aβ monomers were incubated at 37 °C for 3 days to form the Aβ aggregates, and these solutions were stored at −20 °C before use. The same procedure was applied for samples in human serum. We used a Malvern Zetasizer Nano-ZS (Malvern Instruments Ltd.) to assess the hydrodynamic radius of the protein aggregates in the solutions. The particle size analysis was carried out using the refractive index of protein (RI = 1.45) and that of PBS (RI = 1.332) at room temperature (25 °C). The size of the Aβ aggregates was examined for three concentrations of Aβ solutions ranging from femtomolar to nanomolar. We report values on the basis of the highest intensity acquired. The number of aggregates in a certain sample solution was calculated by first dividing the molecular weight of the aggregates estimated from the Zetasizer with the Aβ 1–42 weight (*M*_w_ = 4.5 kDa). As we found the number of peptides forming one aggregate, we considered the peptide concentration in the prepared sample solution and calculated the number of aggregates at a given volume of the aggregate solution.

### Surface Characterization of the Membrane

The X-ray photoelectron spectroscopy (XPS) spectra of the membrane were recorded using a KRATOS Analytical AMICUS instrument equipped with an achromatic Al Kα X-ray source (1468.6 eV). The source was operated at a voltage of 10 kV and a current of 10 mA. The elemental narrow scan region was acquired with a step of 0.1 eV. We calibrated the obtained spectra using the reference C 1s at 284.8 eV. A field emission scanning electron microscopy (FESEM) image was acquired using a Nova Nano instrument at an accelerating voltage of 5 kV and 100 000× magnification. Statistical image analysis was performed using ImageJ to determine the membrane pore size.^55^ The segmentation of the FESEM images was processed first by converting the grayscale images into binary (black and white) ones (Figure S24). The scale bar of each FESEM image was used as the reference length scale to determine the pixel size in nanometers. The pore size was obtained by measuring and averaging the pore diameter in pixel units at four different directions in each pore. This procedure was repeated to calculate the average pore size for each membrane by examining at least 200 pores, sampled from multiple FESEM images of the same membrane. Atomic force microscopy (AFM) measurements were performed with a Bruker Dimension Icon SPM in a resonance frequency range of 76–263 kHz with tapping mode.

### Fabrication of the μf-OECT

We adopted a parallel plate OECT configuration in which two identical gate electrodes were sputtered on the top glass substrate and six identical transistor channels were microfabricated on the bottom substrate (Figure 1). The width and length of the gate electrodes were 1000 and 5000 μm, respectively. We used two different gate electrode materials: silver/silver chloride (Ag/AgCl, nonpolarizable) and gold (polarizable). Ten nanometers of chromium (Cr) and 100 nm of gold (Au) were sputtered on the substrates and patterned the metal through a standard lift-off process. We electrochemically deposited Ag/AgCl on gold-coated glass slides by using a two-step deposition: (i) potentiostatic deposition of Ag particles from 0.01 M AgNO_3_ at −0.2 V for 30 s and (ii) the conditioning and coating of AgCl by immersing the Ag-modified gate in 0.1 M NaCl under a bias of 0.6 V for 5 min, followed by washing with deionized (DI) water and drying with a N_2_ gas spray. The OECT channels were microfabricated on glass substrates using standard photolithography and Parylene-C peel-off techniques (see Figure S25 for the process steps). The channels had a width of 100 μm and a length of 10 μm. Before the Parylene-C peel-off step, PEDOT:PSS dispersion containing EG, DBSA, and GOPS was spin-coated (3000 rpm, 45 s) on the substrate. After peeling the sacrificial Parylene-C layer off, we annealed the devices at 140 °C for 1 h and soaked them in DI water overnight. For the accumulation-mode OECTs, the *p*-type, p(g0T2-g6T2), and the *n*-type polymers, p(C_6_NDI-T), were dissolved in chloroform (5 mg/mL) and spin-coated on the substrate at 800 rpm for 45 s. The microfluidic channel was designed with CorelDRAW software and fabricated using a CO_2_ laser (Universal Laser Systems – PLS6.75) by cutting a 30 μm thick pressure-sensitive adhesive (3M, Acrylic Adhesive Transfer-7952MP). The width of the microfluidic channels was kept 1 mm to ensure that both the channels and gates were in contact with the electrolyte. Inlet and outlet ports (0.1 mm in diameter) were drilled using the CO_2_ laser on the top substrate, where the gate electrodes were located, and the microfluidic channel was attached by using alignment marks under a microscope.

The design of the μf-OECT and its integration with the isoporous membrane is shown in Figure 1. Our design contains two separate microfluidic channels, and each of them encompasses three channels gated by one top gate electrode. The microfluidic channels were intentionally separated from each other to avoid electrical and biological crosstalk issues. The surface of the bottom substrate bearing the transistor channels was covered with the isoporous membrane and adhered to the microfluidic channel to present the final form of μf-OECT comprising the isoporous membrane. The sample or the measurement electrolyte (10 μL) was introduced through the tubes inserted into the microfluidic channel to avoid any backpressure issues. An excess amount was collected at the outlet to be used again. As the same membrane covered six different channels separated by two fluidic channels, the design allowed for estimating results from at least three channels exposed to the same sample solution, while the other three channels acted as a reference (if necessary).

### Electrical Characterization of the μf-OECT and Operation of the Biosensor

We recorded the steady-state characteristics of the transistor using a Keithley 2602A Source Meter unit controlled by a customized LabVIEW software. The drain (*V*_D_) and gate (*V*_G_) voltages were applied while the source electrode functioned as the common ground in both circuits. The steady-state measurements of the PEDOT:PSS-based OECTs were conducted by varying *V*_G_ (0–0.6 V, step 0.05 V) and *V*_D_ (0 to −0.6 V, step of 0.05 V), and the drain current (*I*_D_) was obtained simultaneously. For p(g0T2-g6T2), we applied *V*_G_ from +0.2 to −0.6 V, with a step of 0.05 V, and *V*_D_ from 0 to −0.6 V, with a step of 0.05 V. For p(C_6_NDI-T), we applied *V*_G_ and *V*_D_ from 0.2 to 0.6 V, with a step of 0.05 V. In all measurements, PBS (pH 7.4) was used as the measurement electrolyte.

For sensing experiments, 1 μL of the corresponding Aβ solution (in PBS or serum) was injected through one of the microfluidic channels. After an hour of incubation, the channel was rinsed with PBS to remove any nonspecific adsorption and nonbinding molecules. After the washing step, both microfluidic channels were filled with PBS solution. The devices under the second microfluidic channel were used to monitor background effects. This procedure was repeated for each sample solution. In the absence of microfluidics, we placed an 1 mm thick polydimethylsiloxane (PDMS) well with a diameter of 1 cm on top of the isoporous membrane integrated OECTs to confine 100 μL of solution. The sensor response to different Aβ concentrations was determined from the transfer characteristics (*I*_D_ vs *V*_G_). We calculated the relative normalized response (NR) by considering the protein-induced change in *I*_D_ at a single *V*_D_ and *V*_G_, normalized by its value in the blank solution (*I*_Blank_):
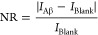
1The limit of detection (LOD) was calculated using the equation:
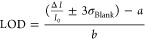
2where  is the average response of the blank sample, *σ*_Blank_ is its standard deviation, and *a* and *b* are the intercept and the slope determined from the calibration curve, respectively.

Electrochemical impedance spectra of the microfluidic and membrane integrated channels were recorded in a two-electrode setup using a potentiostat (Autolab PGSTAT302 Eco Chemie) controlled by the Model NOVA version 1.9 software. Top gate electrodes were used as the reference and counter electrodes. The normalized impedance change was calculated at 10 kHz using the following equation:
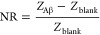
3where *Z*_Aβ_ is the impedance magnitude after Aβ binding, while *Z*_blank_ is the impedance magnitude of the channel covered with the isoporous membrane.

## Supporting Information Available

Figures of FESEM images, size distributions, output and transfer characteristics, transconductance vs *V*_G_ curves, transient characteristics, change in threshold voltage and pinchoff voltage, normalized response of the sensors to Aβ aggregates and to Aβ peptides, electrochemical impedance characteristics, total capacitance, power consumption of the devices under various operating conditions, numerical simulation domain, average electric field intensity and ion concentration on the channel surface, chemical structure, ^1^H NMR spectrum, schematic representation the of PS-*b*-P4VP membrane fabrication, illustration of the fabrication process, and optical microscopy image and discussions of capacitance characteristics of μf-OECTs, numerical simulations, general procedures, synthetic methods, isoporous membrane fabrication, and OECT fabrication (PDF)
