# Supplementary material for: Microfluidic Integrated Organic Electrochemical Transistor with a Nanoporous Membrane for Amyloid-β Detection
Source: ACS Nano. 2021 Mar 30;15(5):8130–41. doi: 10.1021/acsnano.0c09893 (PMC8158856; doi:10.1021/acsnano.0c09893)
Supplement: Supplementary file 1 — nn0c09893_si_001.pdf [file nn0c09893_si_001.pdf]

A Microfluidic Integrated Organic Electrochemical Transistor with a Nanoporous Membrane for Amyloid-β Detection

Anil Koklu[1], Shofarul Wustoni[1], Valentina-Elena Musteata[2], David Ohayon[1], Maximilian Moser[3], Iain McCulloch[3,4], Suzana P. Nunes[2], Sahika Inal[1*]

[1]King Abdullah University of Science and Technology (KAUST), Biological and Environmental Science and Engineering (BESE), Organic Bioelectronics Laboratory, Thuwal 23955-6900, Saudi Arabia.

[2] KAUST, BESE, Advanced Membranes and Porous Materials Center, Thuwal 23955-6900, Saudi Arabia.

[3] University of Oxford, Department of Chemistry, Oxford, OX1 3TA, United Kingdom

[4] KAUST, Physical Science and Engineering Division, KAUST Solar Center (KSC), Thuwal 23955-6900, Saudi Arabia.

[*]Corresponding author: sahika.inal@kaust.edu.sa

Keywords: amyloid-β, organic electrochemical transistor, microfluidics, isoporous membrane, biosensors.

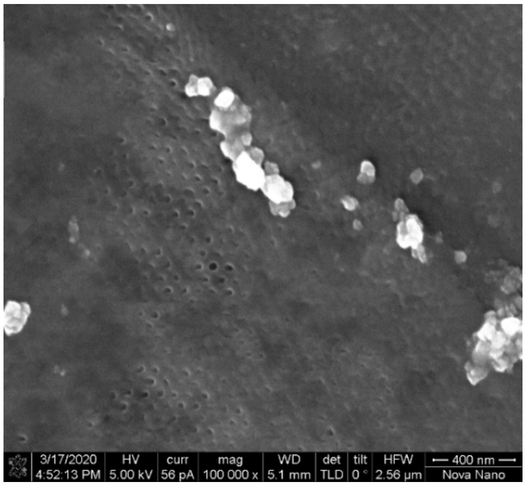

Figure S1. FESEM image of Aβ aggregates captured on the surface of a CR-modified nanoporous membrane.

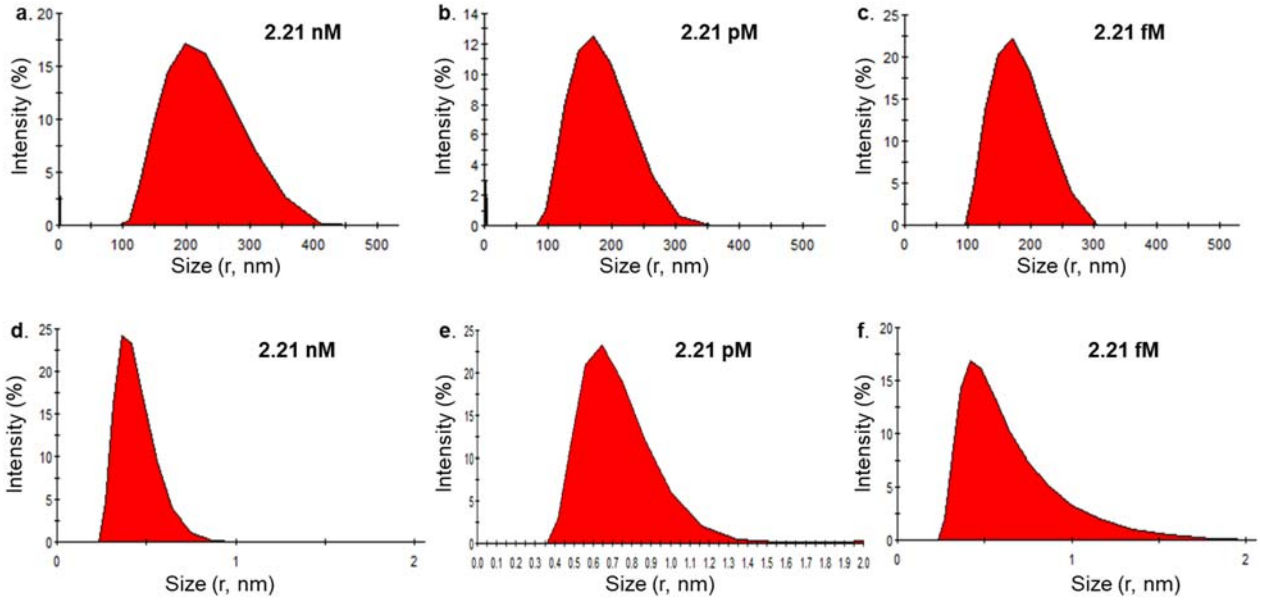

Figure S2. The size distribution of Aβ aggregates (a-c) and Aβ monomers (d-f) at different concentrations. The hydrodynamic radius is estimated based on the empirical mark. The values reported in the text refer to the radii with the peak intensity. We calculated the number of Aβ aggregates in a given sample volume and concentration based on the molecular weight and size estimations.

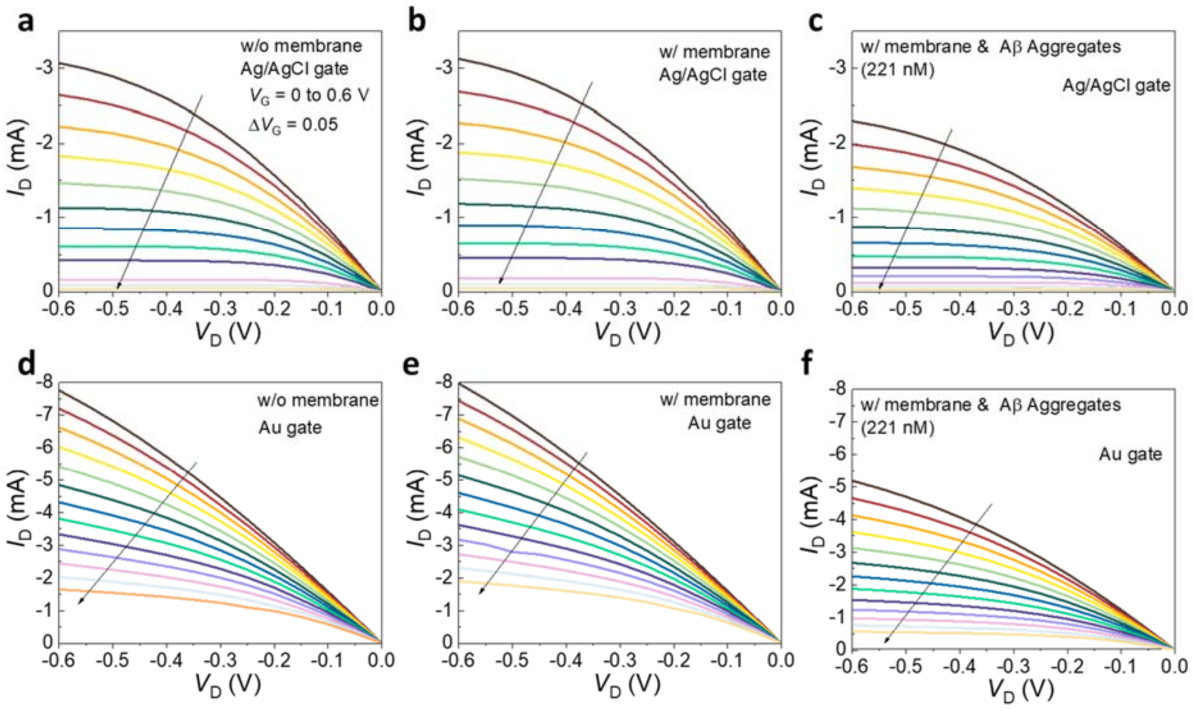

Figure S3. Output characteristics of the PEDOT:PSS µf-OECTs **a)**, **d)** before and **b)**, **e)** after the integration of the CR-functionalized membrane and, **c)**, **f)** after Aβ aggregate binding. The electrolyte was 10 mM PBS, and the gate electrode was Ag/AgCl for a-c and Au for d-f. The arrow direction indicates the increase in V_G magnitude.

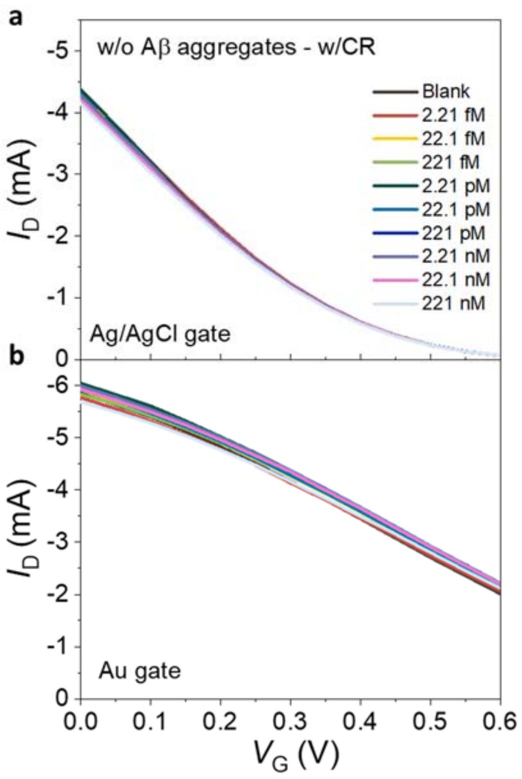

Figure S4. Transfer characteristics of PEDOT:PSS μf-OECTs exposed to blank PBS solution injected several times. The gate electrode is **a)** Ag/AgCl and **b)** Au.

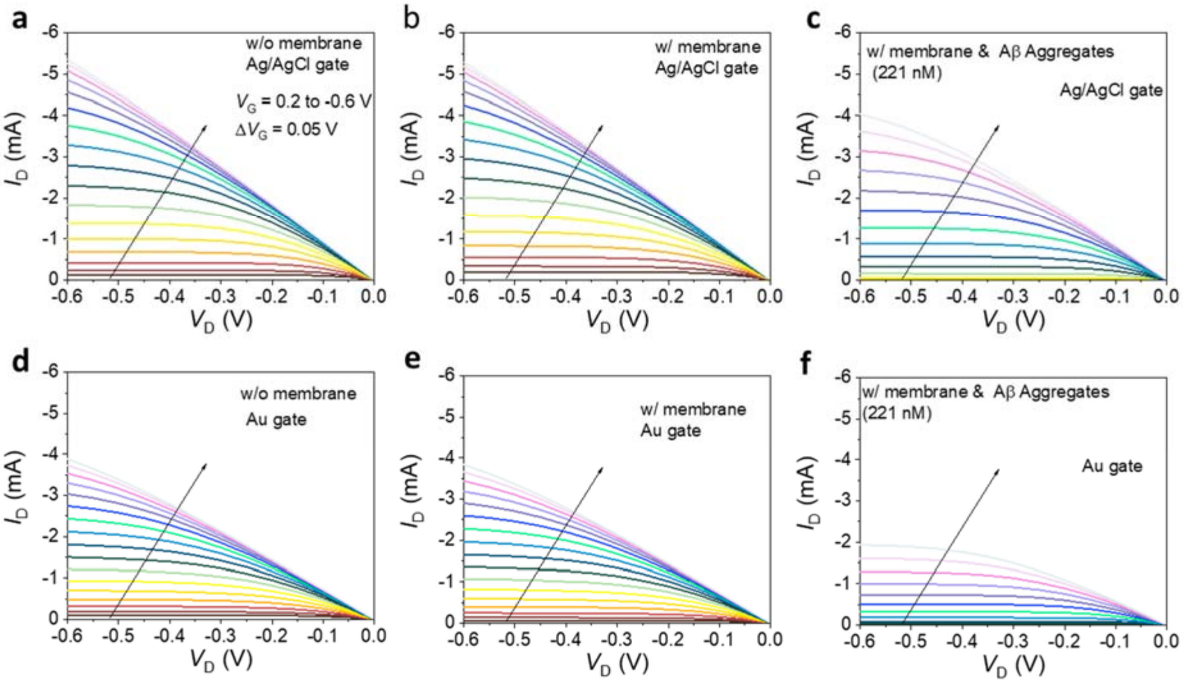

Figure S5. Output characteristics of p(g0T2-g6T2) μf-OECTs **a)**, **d)** before, **b)**, **e)** after the integration of the CR-functionalized membrane and **c)**, **f)** after Aβ aggregate binding. The electrolyte was 10 mM PBS, and the gate electrode was Ag/AgCl for a-c and Au for d-f. The arrow direction indicates the increase in V_G magnitude.

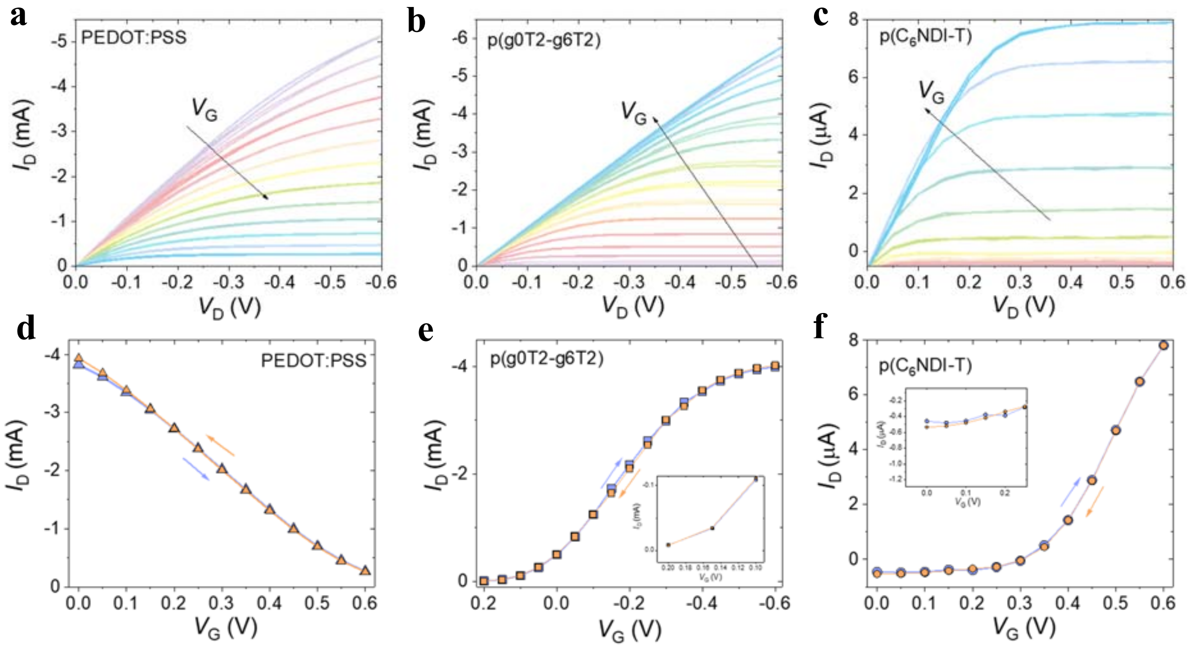

Figure S6. OECT hysteresis: The output curves (**a-c**) and the saturation regime transfer curves (**d-f**) of PEDOT:PSS, p(g0T2-g6T2) and p(C_6NDI-T) devices. The gate voltages stepped in 0.05 V increments and the scan rate was 50 mV/s. The forward scan is shown with the arrows in (a-c). In **e-f**, the insets show the zoomed-in I_D at low V_G. The arrows indicate the scan direction.

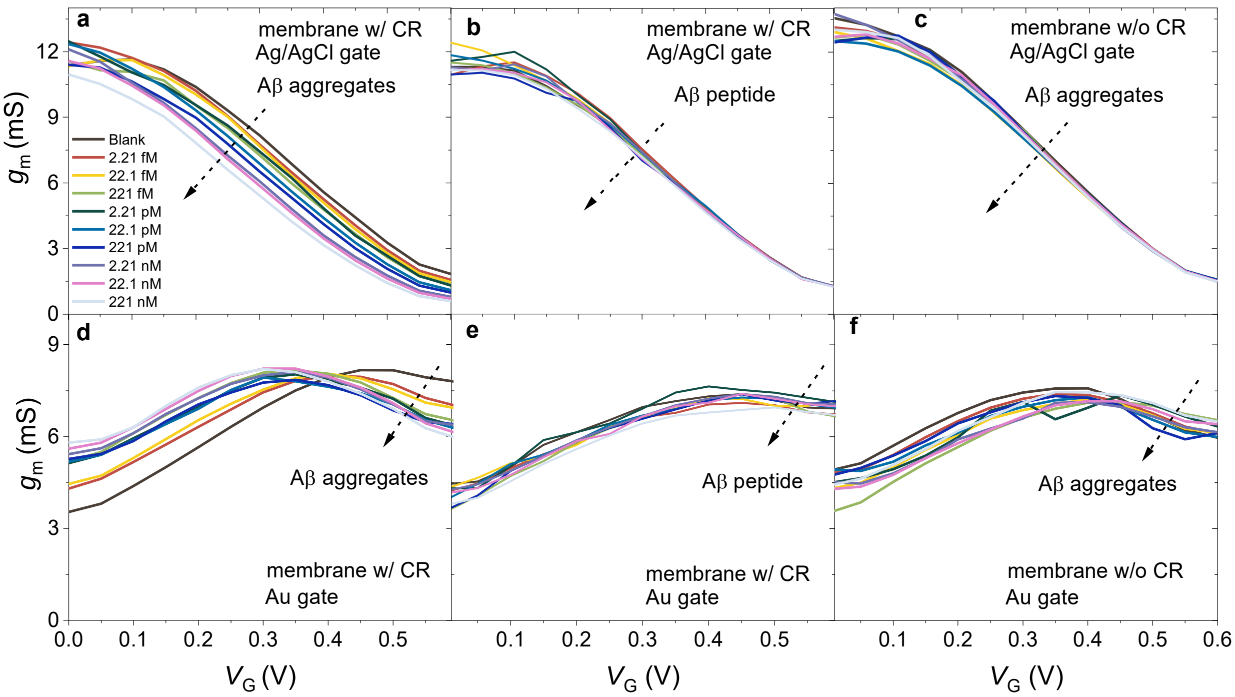

Figure S7. Transconductance *vs.* V_G curves of **a-c)** Ag/AgCl and **d-f)** Au electrode gated μf-OECTs comprising PEDOT:PSS in the channel. The arrows represent the increase in Aβ aggregate (**a** and **d**) and Aβ peptide (**b** and **e**) concentrations in the sample solution from 2.21 fM to 221 nM. The transfer characteristics of the pristine membrane (no CR functionalization) integrated devices are shown in **c)** and **f)**. V_D was -0.6 V.

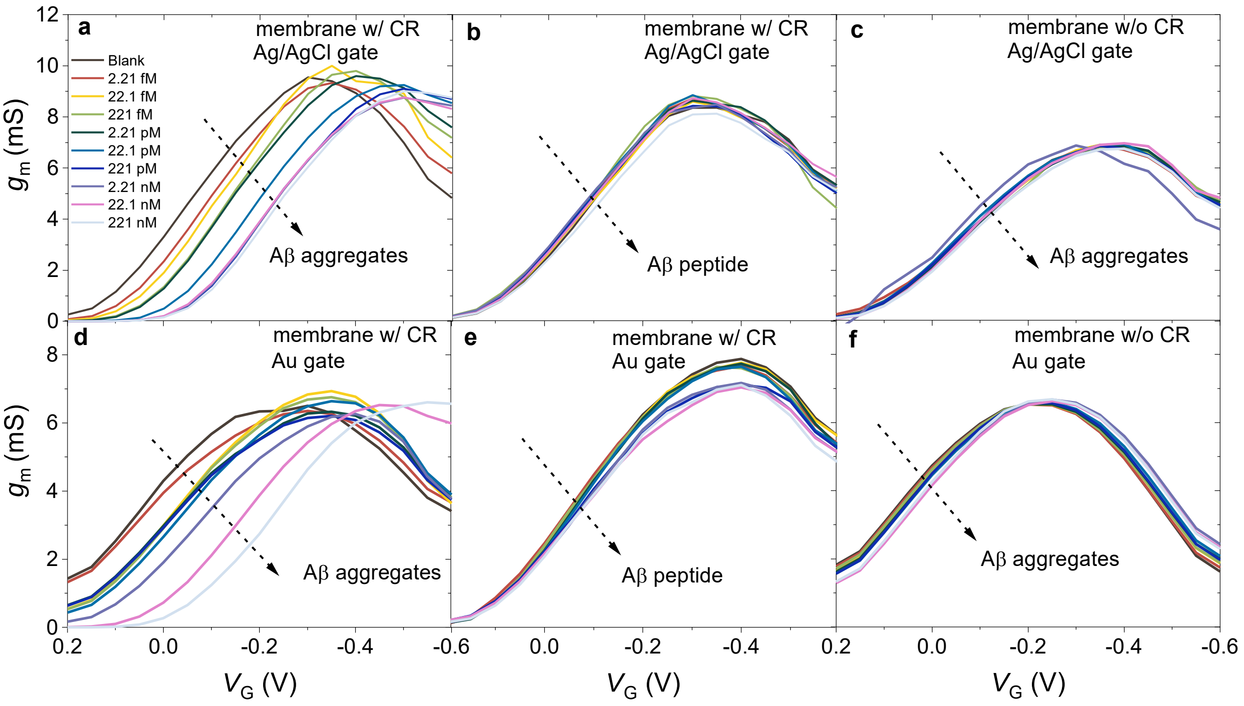

Figure S8. Transconductance *vs.* V_G curves of **a-c)** Ag/AgCl and **d-f)** Au electrode gated µf-OECTs comprising p(g0T2-g6T2) in the channel. The arrows represent the increase in Aβ aggregate (**a** and **d**) and Aβ peptide (**b** and **e**) concentration in the sample solution from 2.21 fM to 221 nM. The transfer characteristics of the pristine membrane (no CR functionalization) integrated devices are shown in **c)** and **f)**. V_D was -0.6 V.

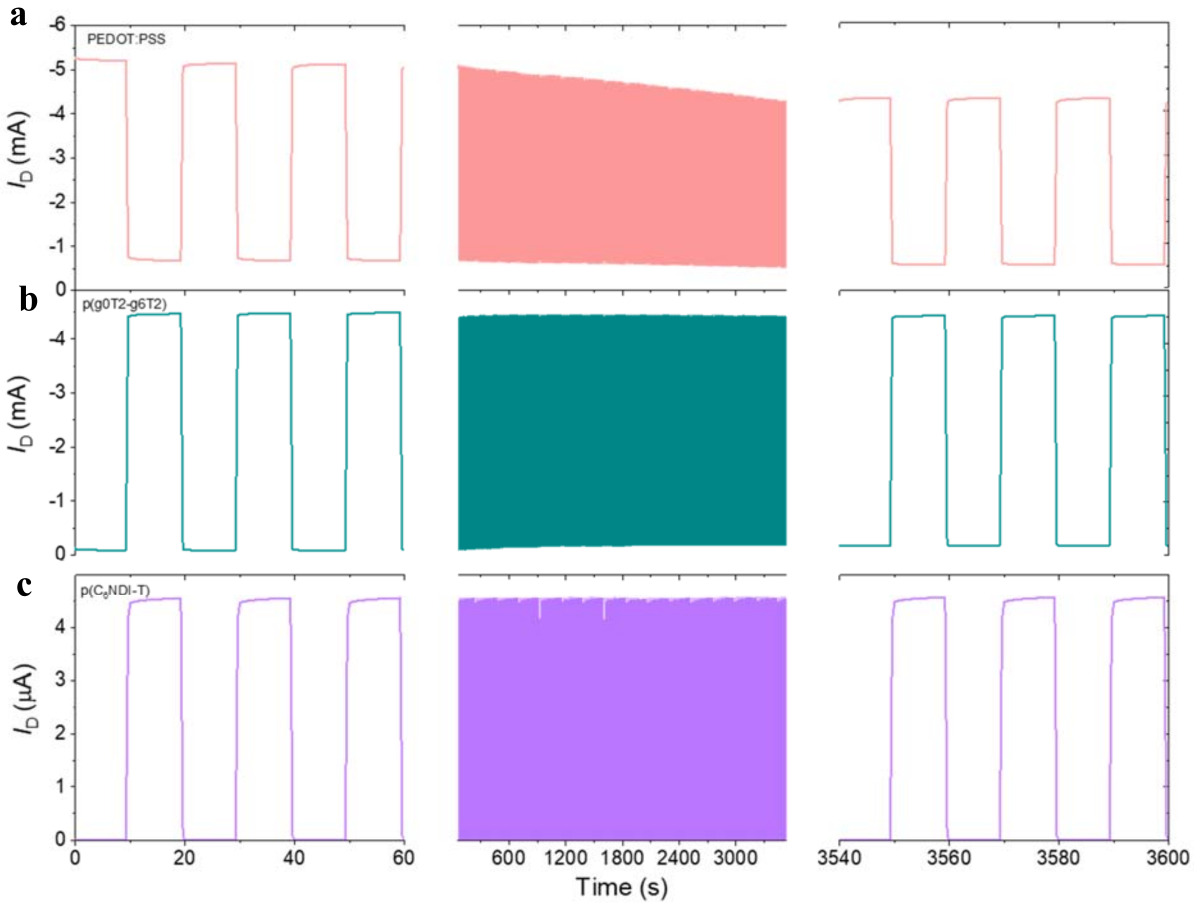

Figure S9. Transient characteristics of PEDOT:PSS **(a)**, p(g0T2-g6T2) **(b)**, and p(C_6NDI-T) **(c)** devices over an hour of continuous ON and OFF biasing (10 seconds each) at the gate electrode. The operation conditions of PEDOT:PSS, p(g0T2-g6T2), and p(C_6NDI-T) are V_D = -0.5 V, V_G = 0.5 V; V_D = -0.5 V, V_G = -0.5 V; and V_D = 0.5 V, V_G = 0.5 V, respectively.

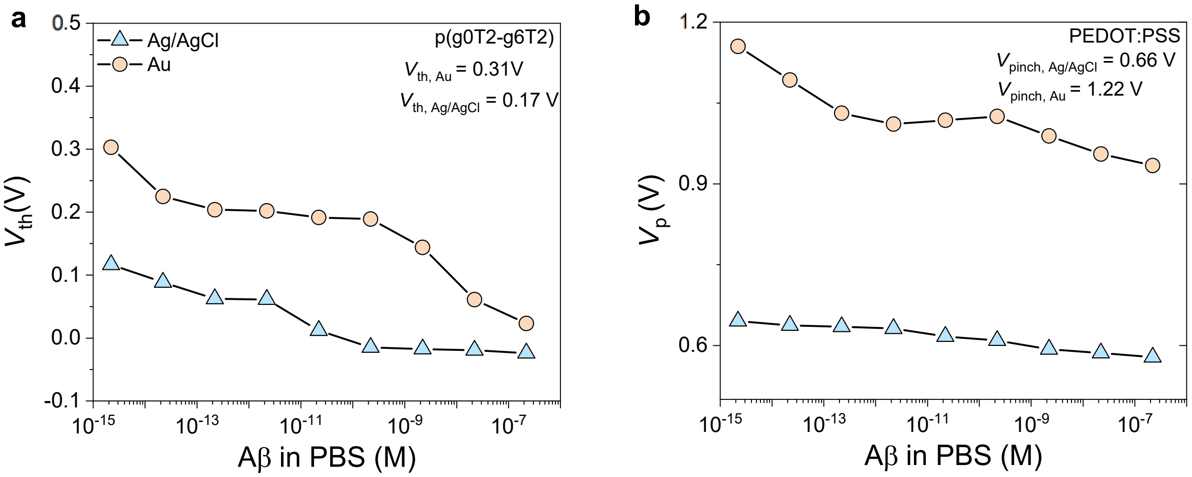

Figure S10. The change in the **a)** threshold voltage (V_{th}) of p(g0T2-g6T2) μf-OECTs and **b)** pinch-off voltage (V_p) of PEDOT:PSS μf-OECTs as a function of Aβ aggregate concentration.

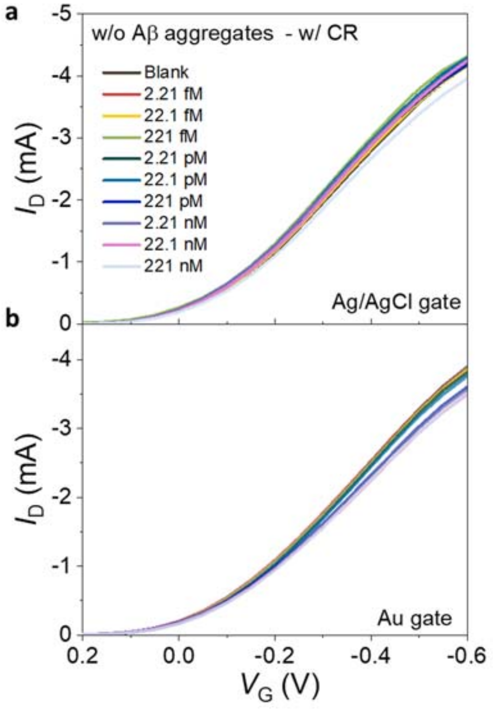

Figure S11. Transfer characteristics of p(g0T2-g6T2) μf-OECT exposed to blank PBS solution injected several times. The gate electrode is **a)** Ag/AgCl and **b)** Au.

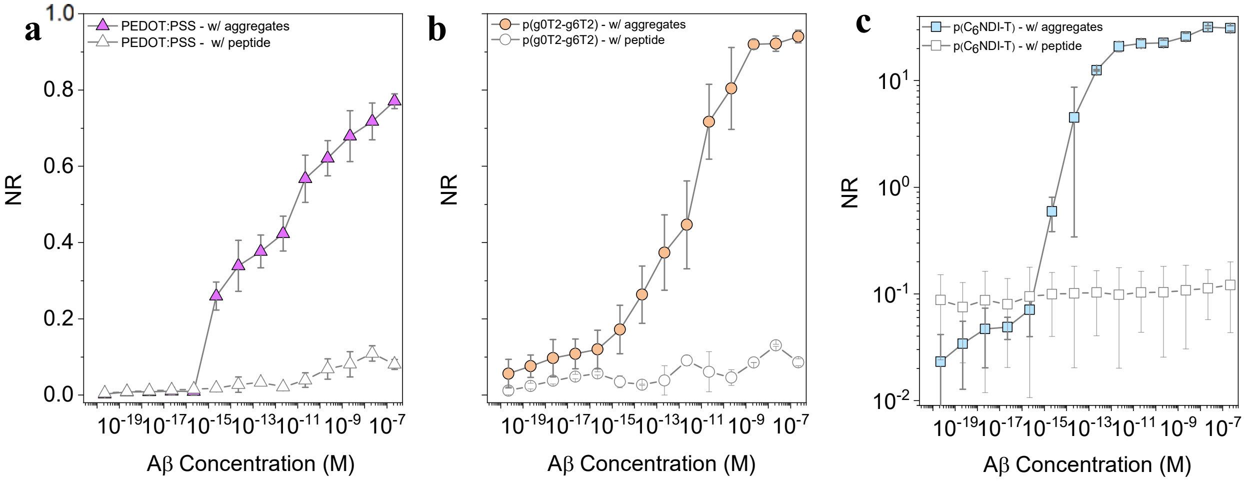

Figure S12. Normalized response (NR) of the sensors to Aβ aggregates (filled symbols) and to Aβ peptides (hollow symbols) with channels made of **a)** PEDOT:PSS, **b)** p(g0T2-g6T2) and **c)** p(C_6NDI-T). NR was calculated at V_G = 0.6 V for PEDOT:PSS, V_G = -0.1 V for p(g0T2-g6T2) while V_D was -0.6 V. For the *n*-type channel, NR was reported at V_G = 0.3 V and V_D = 0.2 V. All devices were gated with Ag/AgCl and error bars represent the SD from at least 3 different channels. In c), a log scale was used to better visualize the response at the low concentration range.

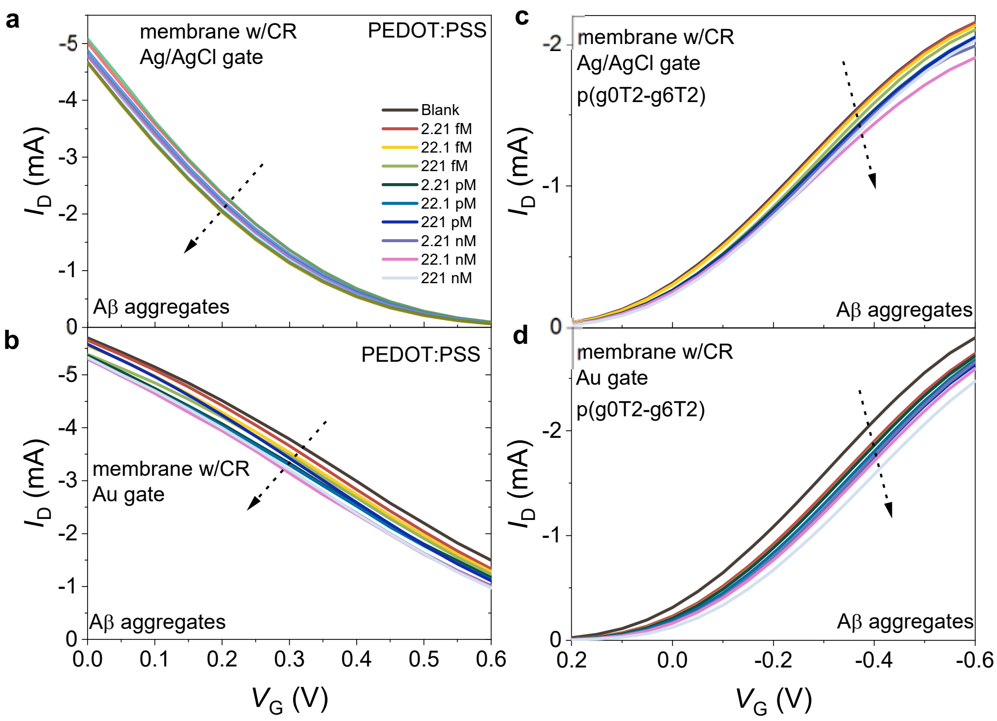

Figure S13. Transfer characteristics of OECT sensors recorded using a PDMS chamber. The channel is PEDOT:PSS in **a-b** and p(g0T2-g6T2) in **c-d**. The gate electrode is **a)**, **c)** Ag/AgCl and **b)**, **d)** Au.

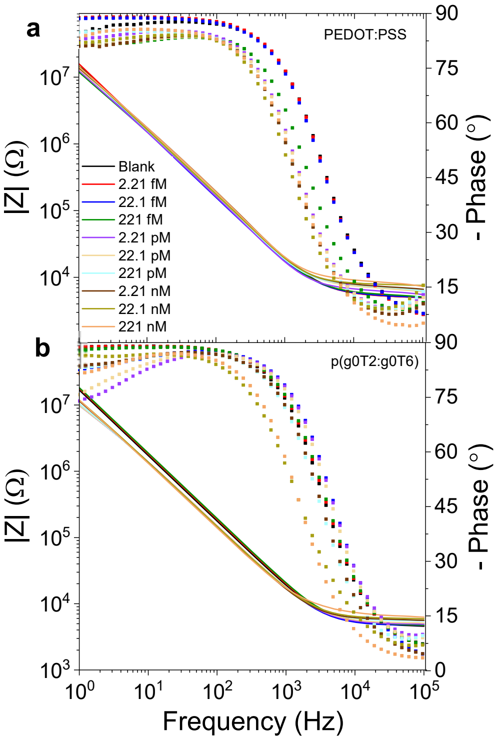

Figure S14. Electrochemical impedance characteristics of **a)** PEDOT:PSS and **b)** p(g0T2-g6T2) channels upon incubation with Aβ molecules at various concentrations. The measurements were carried out at open circuit potential.

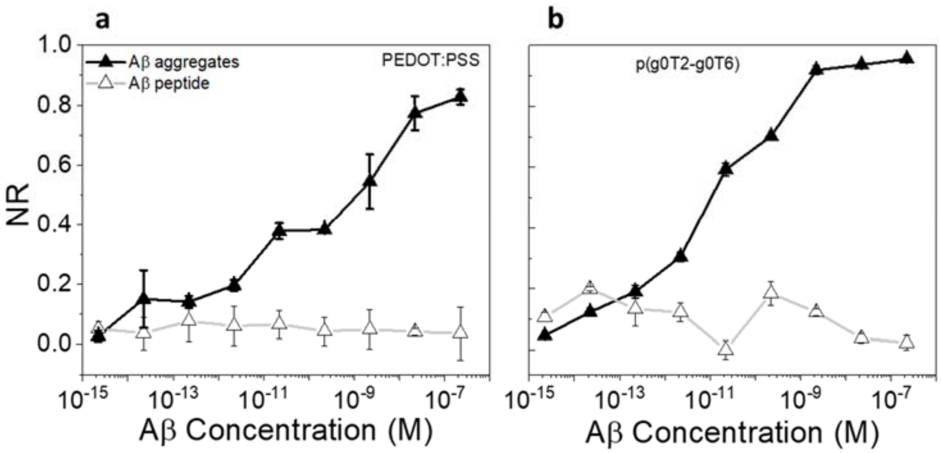

Figure S15. NR of **a)** PEDOT:PSS and **b)** p(g0T2-g6T2) μf-OECTs to Aβ aggregates or Aβ peptide spiked at various concentrations in human serum. NR was determined at V_G = 0.6 V for PEDOT:PSS and V_G = -0.1 V for p(g0T2-g6T2) devices gated with Au. Error bars represent the SD from at least 3 different channels.

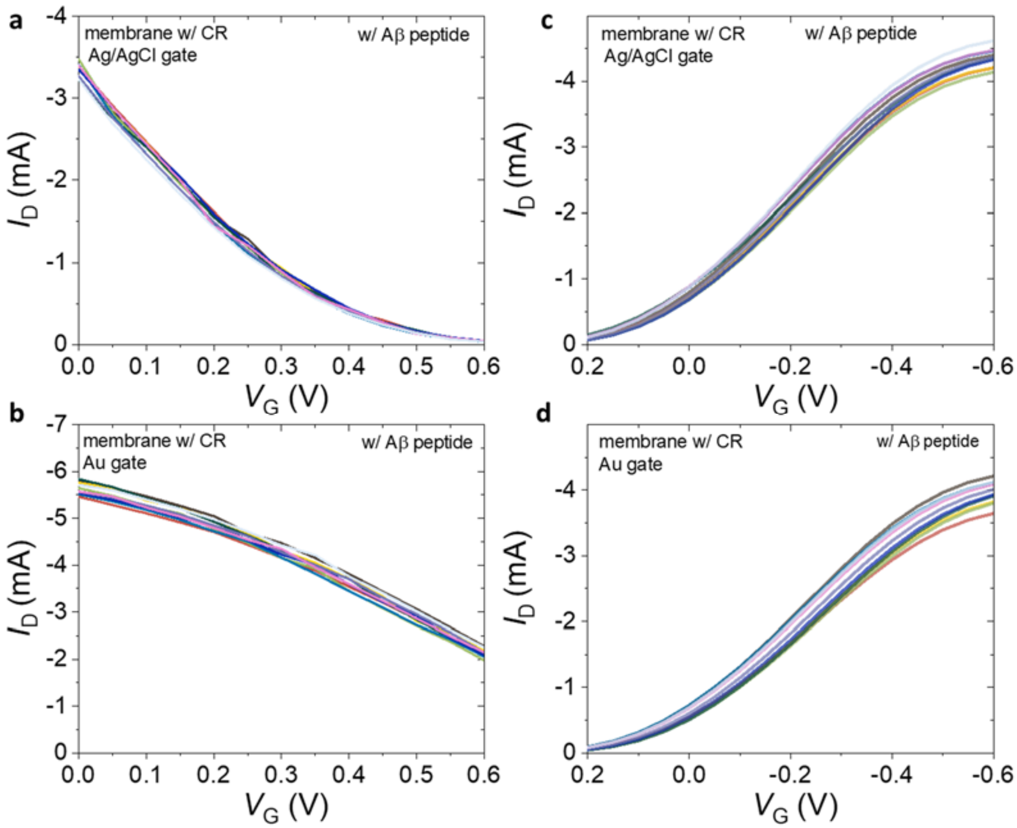

Figure S16. Transfer characteristics of OECT sensors as they were exposed to human serum samples containing various concentrations of Aβ peptide (different colors indicate the concentrations from 2.21 fM to 22.1 nM). The channel was PEDOT:PSS in **a-b** and p(g0T2-g6T2) in **c-d**. The gate electrode is **a)**, **c)** Ag/AgCl and **b)**, **d)** Au.

Capacitance characteristics of µf-OECTs

To gain physical insight into the capacitance changes of the OECT interfaces, we analyzed the transfer characteristics of PEDOT:PSS based devices exposed to various Aβ concentrations using Bernards and Malliaras model. The model relates the channel current to the capacitance of the system as the following:[1]

$$I_D = -\frac{G}{2V_p}\left(V_p - V_E\right)^2 \; if \; V_D < V_E - V_p \tag{1}$$

$$I_D = -\frac{G}{V_p}\left(V_p - V_E + \frac{V_D}{2}\right)\ if\ V_D \geq V_E - V_p$$

where

$$G = \frac{wt}{L} q\mu p_0$$
$$V_p = \frac{qp_0 v}{C_{tot}} \quad (2)$$

where G is the conductance, V_p is the pinch-off voltage, V_E is the voltage sensed in the electrolyte after the voltage drop at the gate electrode/electrolyte interface, V_D is the drain voltage, w, t, L, and v are the width, thickness, length and volume ($v = w\ t\ L$) of the channel, respectively. q is the elementary charge, $\mu = 2\ cm^2 Vs^{-1}$ is the hole mobility of PEDOT:PSS, $p_0 = 1.8\ \times 10^{20}\ cm^{-3}$ is the intrinsic hole density of PEDOT:PSS. C_{tot} is the overall capacitance, which accounts for the series of the electric double-layer capacitances at the gate–electrolyte interface (C_G) and the channel–electrolyte interface ($C_c = C_v\ v$), where $C_v = 40\ Fcm^{-3}$ is the volumetric capacitance of PEDOT:PSS . We plotted square root of I_D ($\sqrt{I_D}$) as a function of the gate voltage (V_G), from the data in Figure 3 obtained for different concentrations of Aβ. The point at which the slope of the $\sqrt{I_D}$ intercepts the x axis gives the V_p, and in turn C_{tot} (equation 2). The extracted C_{tot} as a function of Aβ concentrations is depicted in Figure S17.

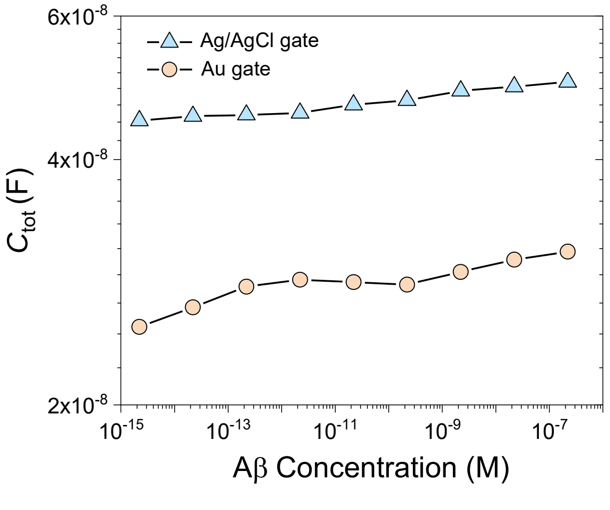

Figure S17. The total capacitance of the PEDOT:PSS channel gated with Ag/AgCl or Au electrode.

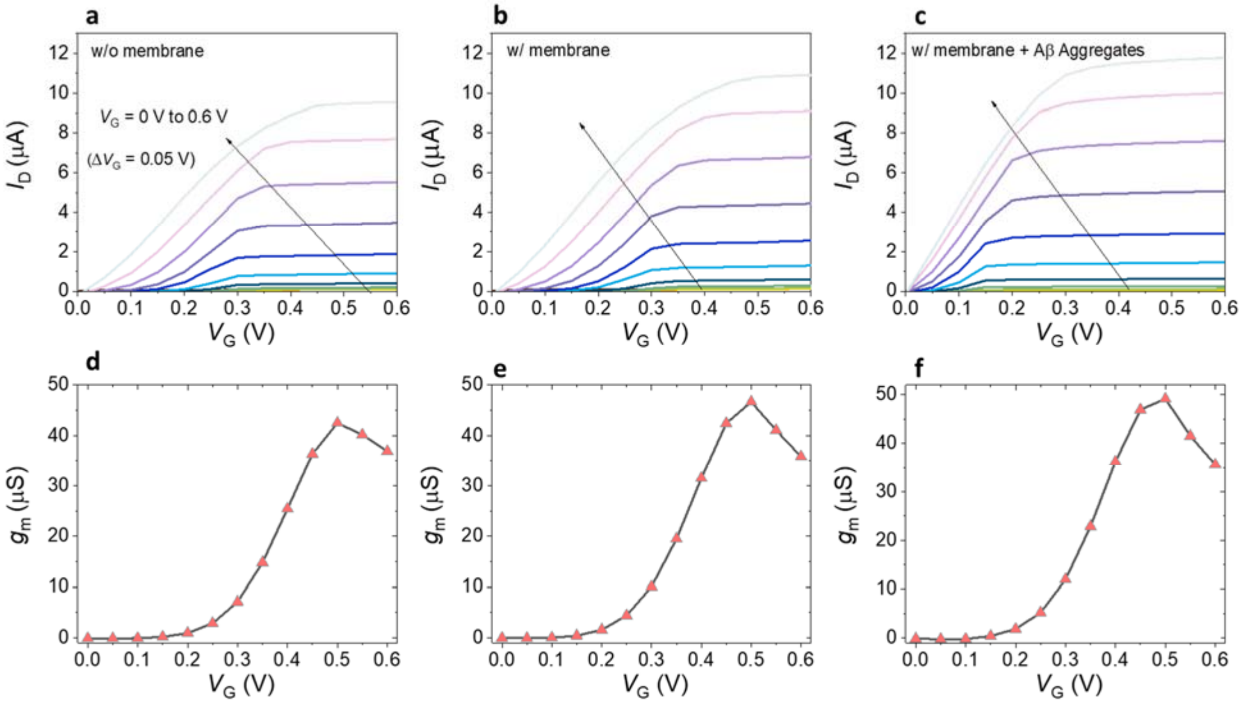

Figure S18. Output characteristics of the *n*-type p(C_6NDI-T) OECTs before **a)** and after **b)** integration of the CR-functionalized membrane and, **c)** after binding of Aβ aggregates (221 nM). V_G increased from 0 V to 0.6 V with a step of 0.05 V. The electrolyte was 10 mM PBS and the gate electrode was Ag/AgCl. The corresponding transconductance *vs.* V_G plots calculated at V_D = 0.6 V is shown in **d-f**.

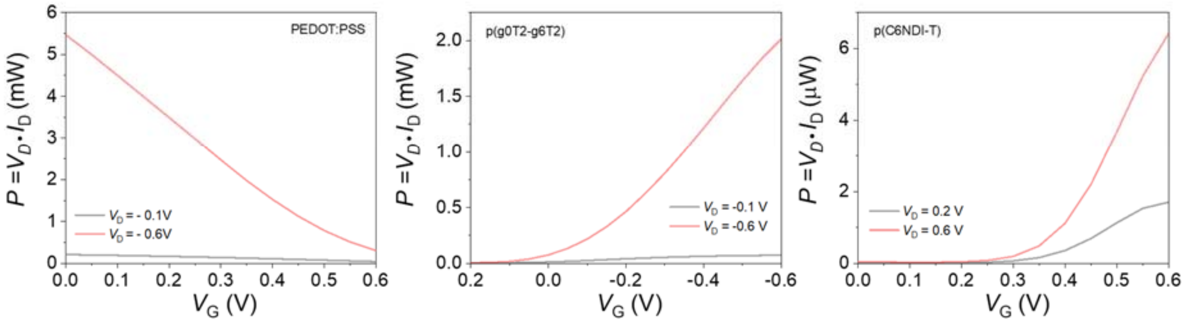

Figure S19. The power consumption of the devices under various operating conditions. I_G is negligible for all devices.

Numerical Simulations

The coupled Poisson-Nernst-Planck (PNP) equations incorporate ion transport under the electric field gradients for a dilute, completely dissociated ionic solution. The equations were solved using COMSOL Multiphysics 5.5. Since the microfluidic channel's width is much larger than its height, we considered ion accumulation at the OECT channel surface in a rectangular two-dimensional system. A finer mesh was distributed near the OECT channel surface to resolve ion concentration and capture the large electric field gradients. For all cases, the initial cation and anion concentrations were set to bulk concentration, and initial potential was set to zero such that the system was at rest at t=0. From t=0 onward, a time-stepping routine combined with the direct-solving method (MUMPS) was used to compute the time-dependent ion accumulation behavior at an applied voltage for each channel material. Note that our numerical model does not consider ions penetrating the channel. We only simulated the fluidic part where the electric field and transport equations were solved to predict the system behavior. The governing equations for the electrode/electrolyte system are dictated by Gauss' law (S1a and S1c) and the charge conservation equation (S1b):

$$\rho_q = \nabla \cdot (\varepsilon \boldsymbol{E}) \tag{S1a}$$

$$\frac{\partial \rho_q}{\partial t} + \nabla \cdot (\sigma \boldsymbol{E}) = 0 \tag{S1b}$$

$$\nabla \times \boldsymbol{E} = 0 \tag{S1c}$$

where ρ_q is the space charge density, ε is the permittivity of the fluid, $\boldsymbol{E}$ is the electric field, and σ is the electrical conductivity of the medium. We neglected the contribution of the convective term and magnetic field effect by assuming a quasi-electrostatic field.

The simulation domain is depicted in Figure S20a. The concentration of ions inside the medium (Na^+ and Cl^-) are described by the Nernst-Planck equation:

$$\frac{\partial c_i}{\partial t} + \nabla \cdot \left(-D_i \nabla c_i - \frac{z_i D_i F c_i}{RT} \nabla \psi \right) = 0 \quad \text{(S2)}$$

where D_i is the diffusion coefficient of species i and ψ is the electric potential. The electric field in the media is obtained by solving Poisson equation:

$$\nabla \cdot (\varepsilon \boldsymbol{E}) = F \sum_i z_i c_i \quad \text{(S3)}$$

where, $\boldsymbol{E}$ is the electric field ($\boldsymbol{E} = -\nabla \psi$).

The simulation parameters and the boundary conditions are as follows. The operating temperature was 300 K and diffusion coefficients of Na^+ and Cl^- ions were fixed at 1.33×10^{-9} m^2/s and 2.30×10^{-9} m^2/s, respectively. The relative permittivity of medium was considered to be 78. All the boundaries were fixed as insulating boundary condition, except the gate and the channel which were excited at a constant voltage (Figure S20a). Once a nanopore was clogged by the Aβ aggregate, an extra floating potential was introduced, which enhanced or hindered ion motion towards the OECT channel. The surface potential of the Aβ aggregate was measured as 15 mV. We thus added an external constant potential layer right above the channel. The bottom boundary was considered ideally polarizable without Faradic processes, so the ionic flux vanished at these boundary conditions. ($\boldsymbol{n} \cdot \boldsymbol{J}_i = 0$), while all the other boundaries were subject to bulk ionic concentrations. The initial cation and anion concentrations were set to the bulk concentrations and the initial potential was set to zero. The domain height was large enough so that the current did not change with further increase in the domain size.

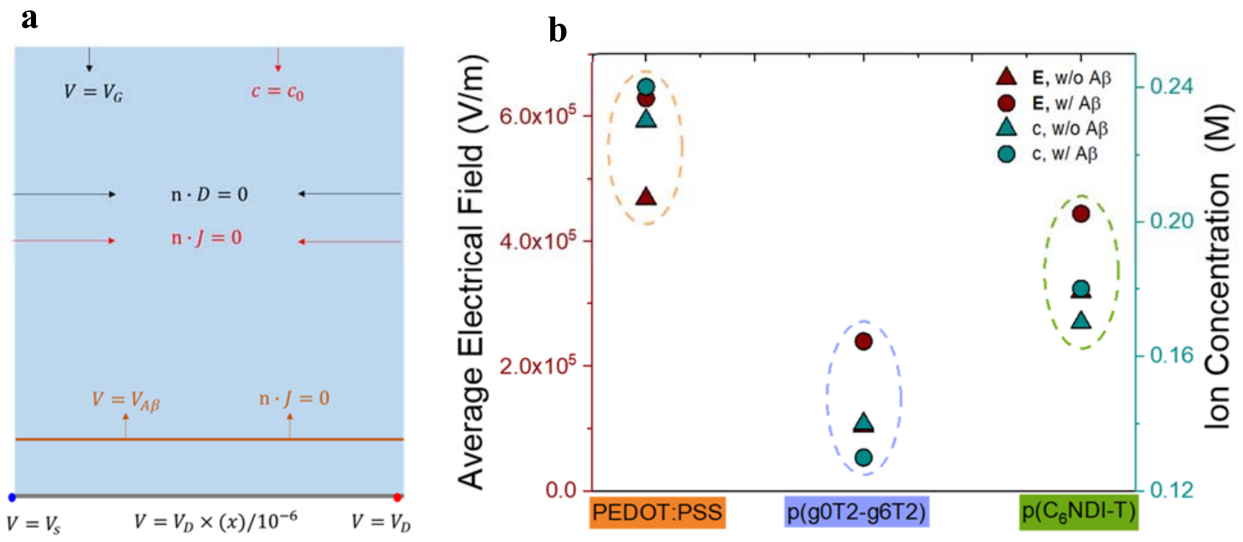

Figure S20. a) Numerical simulation domain along with the boundary conditions for the electrostatic and transport of ionic species physics. **b)** The average electric field intensity and ion concentration on the channel surface. The numerical simulations were performed at V_G = 0.6 V for PEDOT:PSS and V_G = -0.1 V for p(g0T2-g6T2) devices. V_D was -0.6 V in these measurements. For p(C_6NDI-T), V_G was 0.3 V and V_D was 0.2 V.

Synthesis of *n*-type polymer, p(C_6NDI-T)

General Procedures

All reagents were purchased from Sigma-Aldrich or Acros Organics and were used without any further purification. Reactions were conducted in oven dried flasks and under an inert atmosphere of nitrogen. ^{1}H NMR spectroscopy was conducted on Bruker AV-400 UltraShield spectrometers (400 MHz) at 298 K and using chloroform-*d* ($CDCl_3$) as solvent. Chemical shifts (δ) are expressed in parts per million (ppm) downfield from tetramethylsilane (TMS) and are referenced to chloroform (δ_H 7.26). Multiplicities are indicated as singlet (s) or multiplet (m).

Gel permeation chromatography (GPC) was conducted on an Agilent Technologies 1200 series (40 °C, $CHCl_3$) that was calibrated against narrow molecular weight polystyrene standards to extract number average molecular weights (M_n) and dispersities ($Đ$).

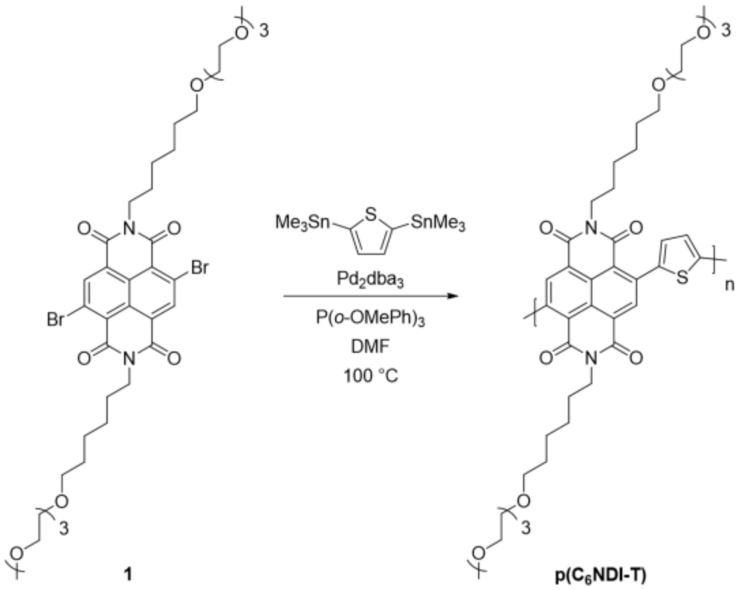

Figure S21. Chemical structure of *n*-type polymer, p(C_6NDI-T).

Synthetic Methods

Compound **1** was synthesized according to the literature. [2] Compound **1** (104.8 mg, 0.1146 mmol, 1.000 eq.), 2,5-bis(trimethylstannyl)thiophene (47.2 mg, 0.115 mmol, 1.00 eq.), tris(dibenzylideneacetone)dipalladium(0) (2.00 mg, $2.19 \cdot 10^{-3}$ mmol, 0.0192 eq.) and tris(*o*-methoxyphenyl)phosphine (3.08 mg, $8.74 \cdot 10^{-3}$ mmol, 0.0763 eq.) were dissolved in 2.3 mL of anhydrous N,N-dimethylformamide and the resulting mixture degassed with nitrogen for 10 min. The mixture was then heated to 100 °C overnight. The polymer was end-capped according to the literature.[3] After cooling to room temperature, the crude polymer solution was precipitated into a 1:1 v/v mixture of hexane:ethyl acetate and purified by successive Soxhlet extractions in hexane, acetone, ethyl acetate, methanol and chloroform. The product was collected in the chloroform fraction. Excess solvent was removed under reduced pressure. The final product was collected by reprecipitating the polymer into a 1:1 v/v mixture of hexane:ethyl acetate and collecting the polymer by suction filtration. p(C_6NDI-T) was obtained as a dark blue solid (82 mg, 86% yield). ^{1}H nuclear magnetic resonance (NMR) (400 MHz, $CDCl_3$, δ): 9.00 (broad s, 2H), 7.50 (broad s, 2H), 4.21 (broad s, 4H), 3.71 – 3.62 (m, 16H), 3.62 – 3.54 (m, 8H), 3.51 – 3.45 (m, 4H), 3.42 – 3.36 (m, 6H), 1.85 – 1.70 (m, 4H), 1.55 – 1.38 (m, 12H); ; GPC ($CHCl_3$, 40 °C) M_n = 18.3 kDa, $Đ$ = 1.99.

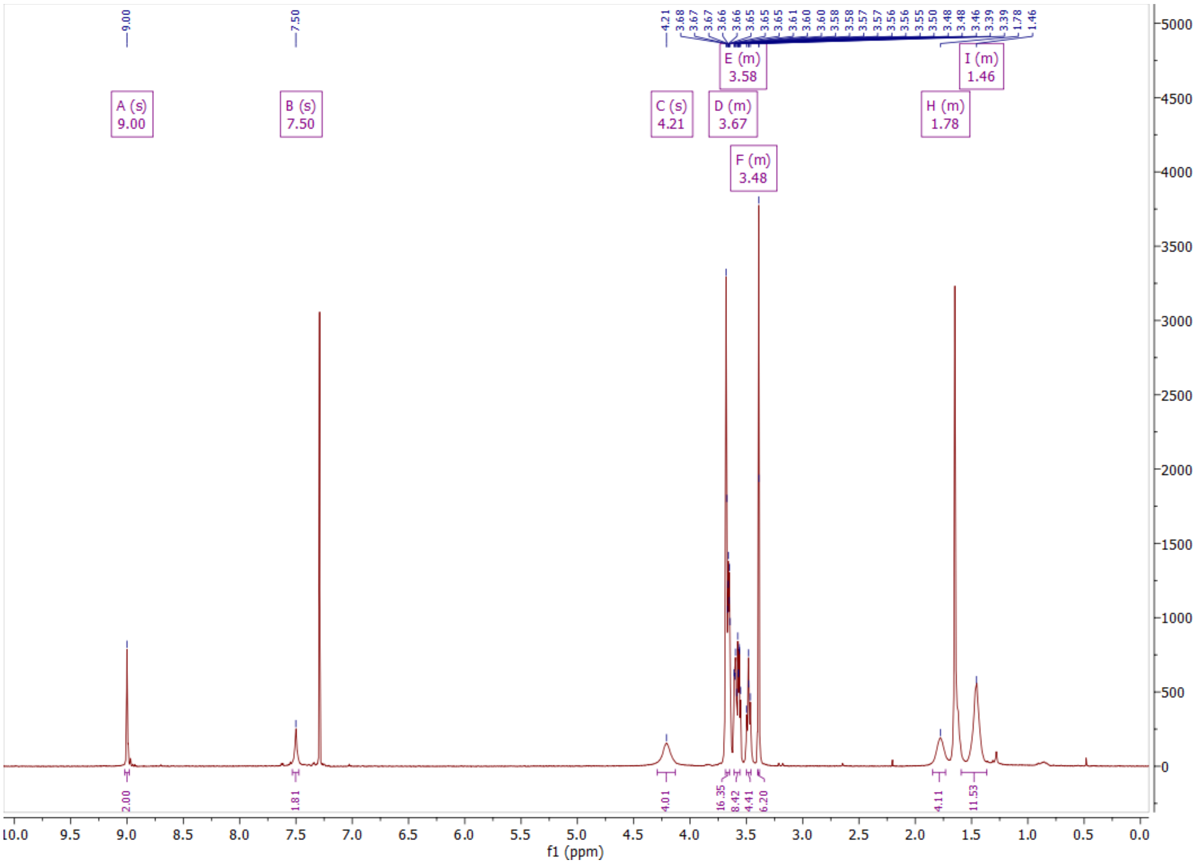

Figure S22. ^{1}H NMR of *n*-type polymer, p(C_6NDI-T).

Isoporous membrane fabrication

The membranes were prepared from a 18% of PS-*b*-P4VP block copolymer solution in a ternary solvent mixture with 24% dimethylformamide, 42% 1,4 dioxane and 16% acetone (all in weight percent). We stirred this solution at room temperature for 24h and then cast it on a glass plate using a doctor blade with a gap of 200 µm. Following 10 s solvent partial evaporation, we immersed the cast solution layer in a water precipitation bath. Subsequently to the solvent-water exchange, we stored the membrane for 24 in deionized water to remove any solvent traces. We obtained an asymmetric membrane with approximatively 40 nm regular pores on the top layer formed by block-copolymer self-assembly in mixed solvents and phase separation in water. The larger pores in the membrane bulk are formed by spinodal decomposition promoted by the solvent-non solvent exchange after immersion in the water bath.

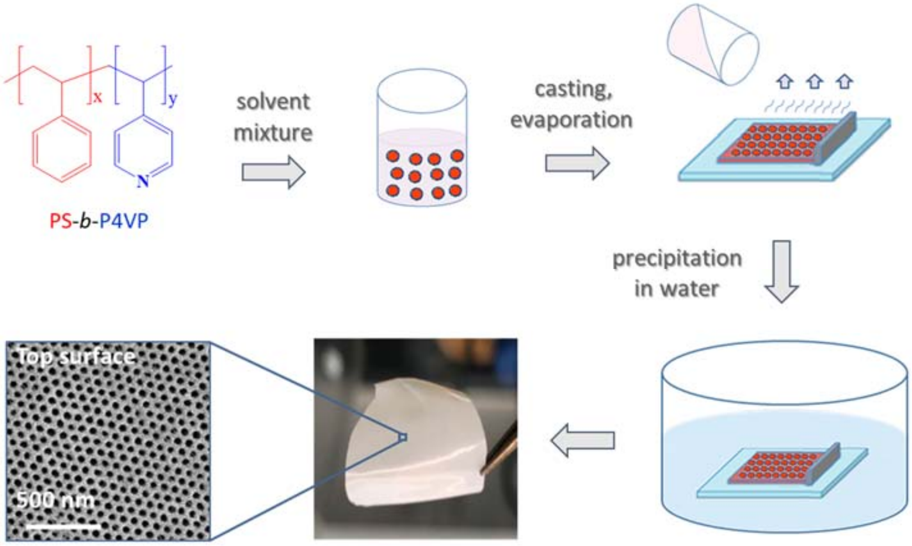

Figure S23. Schematic representation the of PS-*b*-P4VP membrane fabrication.

a) b) c) d)

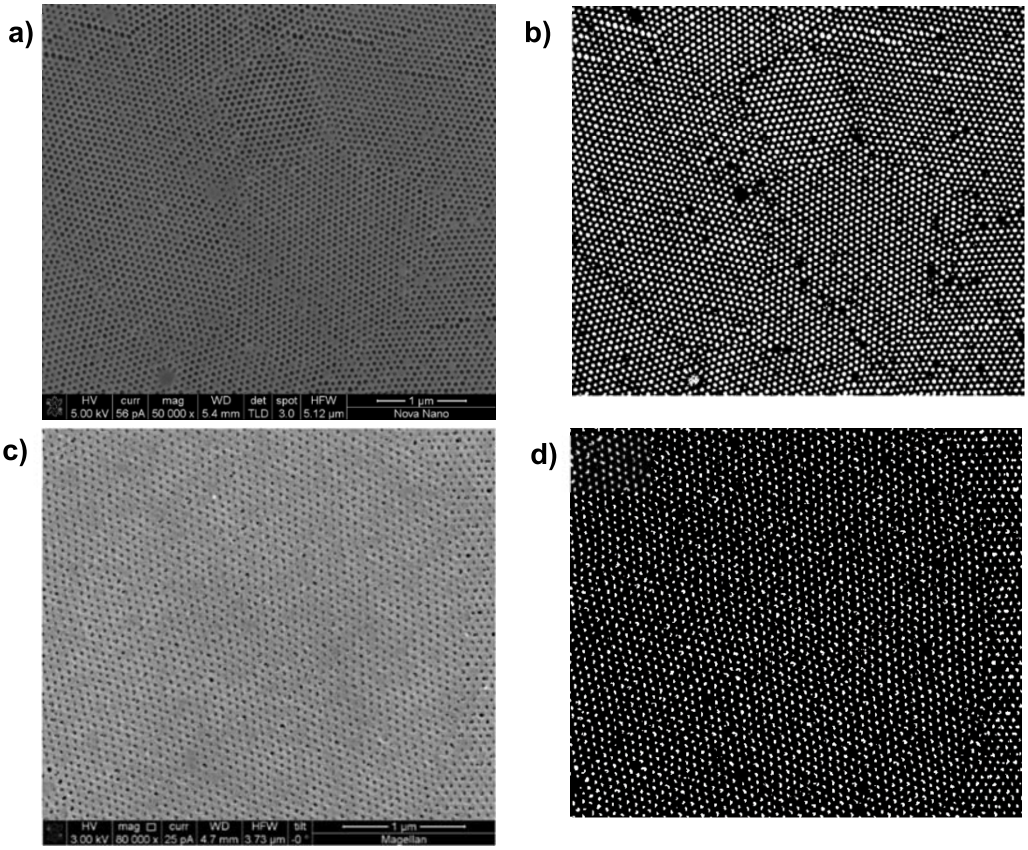

Figure S24. FESEM images of the nanoporous membrane **a)** before and **c)** after CR-functionalization, along with their corresponding binary image versions in **b)** and **d)**.

Organic electrochemical transistor (OECT) fabrication

The OECT fabrication starts with sputtering to deposit chromium (10 nm)/gold (100 nm) on glass wafers. We patterned the metal *via* a lift-off process using a bilayer resist structure (S1813 photoresist, Microchemicals GmbH; LOR 5B MicroChem Corp. Westborough, MA). We performed lift-off in appropriate solvents followed by the encapsulation of the wafer in Parylene C *via* vaporization of the dimer (PDS 2010 Labcoater 2, Specialty Coating Systems, Indianapolis, IN). We spin-coated an anti-adhesion layer on the encapsulated wafer for the deposition of a sacrificial Parylene C layer, which allows for the patterning of the polymer in the channel. The metal contacts and channels were exposed using reactive ion etching with O_2 (Plasma lab 100 - ICP 380, Oxford Instruments). The polymers were spin-coated on the substrates and parylene-C was peeled off to complete the device.

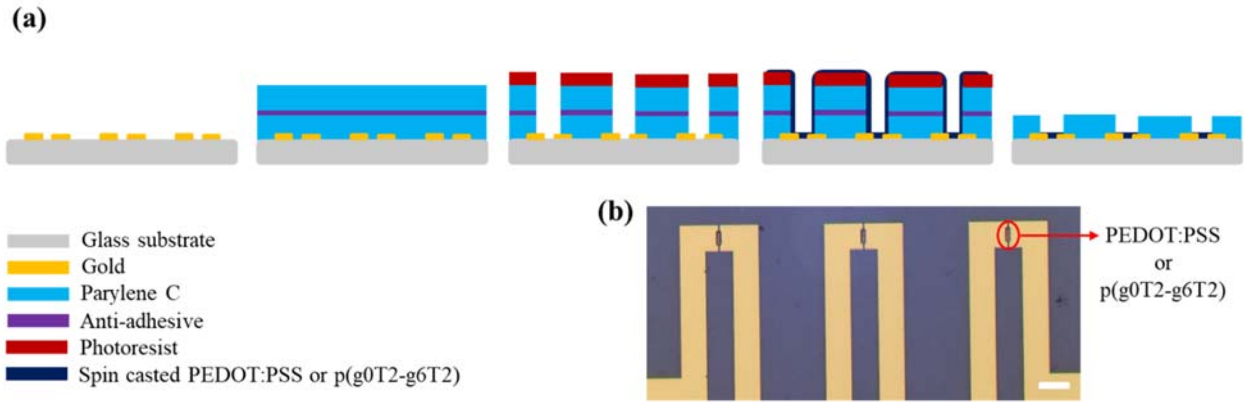

Figure S25. Fabrication of the OECT channels *via* photolithography and Parylene C peel off. **a)** Illustration of the fabrication process starting from the deposition of gold electrode and interconnects until the patterning of the polymer film spin casted in the channels. **b)** Optical microscopy image of the channels. Each channel has a width (*W*) of 100 μm and length (*L*) of 10 μm. Scale bar is 200 μm.

References

1. Bernards, D. A.; Malliaras, G. G., Steady-State and Transient Behavior of Organic Electrochemical Transistors. *Adv. Funct. Mater.* **2007,** *17* (17), 3538-3544.
2. Ohayon, D.; Savva, A.; Du, W.; Paulsen, B. D.; Uguz, I.; Ashraf, R. S.; Rivnay, J.; McCulloch, I.; Inal, S., Influence of Side Chains on the *N*-Type Organic Electrochemical Transistor Performance. *ACS Appl. Mater. Interfaces* **2021,** *13*, 4253-4266.
3. Giovannitti, A.; Rashid, R. B.; Thiburce, Q.; Paulsen, B. D.; Cendra, C.; Thorley, K.; Moia, D.; Mefford, J. T.; Hanifi, D.; Weiyuan, D., Energetic Control of Redox-Active Polymers toward Safe Organic Bioelectronic Materials. *Adv. Mater.* **2020,** *32* (16), 1908047.
